# Human Mucin-2–Producing Colonic Goblet-Like Cells Secrete the Chemokine CXCL8 by Activating Multiple Proinflammatory Pathways in Response to *Entamoeba histolytica*

**DOI:** 10.1016/j.ajpath.2025.02.008

**Published:** 2025-03-22

**Authors:** Ariel Kim, Hayley Gorman, France Moreau, Mackenzie McManus, Antoine Dufour, Kris Chadee

**Affiliations:** ∗ Department of Microbiology, Immunology, and Infectious Diseases, Cumming School of Medicine, University of Calgary, Snyder Institute for Chronic Diseases, Calgary, Alberta, Canada; † Department of Physiology and Pharmacology, Cumming School of Medicine, University of Calgary, Snyder Institute for Chronic Diseases, Calgary, Alberta, Canada

## Abstract

The mucus layer produced by highly stressed goblet cells forms a protective shield in the gut to protect the underlying mucosal epithelial cells from external threats. Hypersecretion and depletion of mucin-2 (MUC2) mucin from goblet cells is characteristic of symptomatic *Entamoeba histolytica* infections. It was hypothesized that MUC2 depleted goblet cells could mount a second line of innate host defense by producing proinflammatory cytokines. To investigate this, whether *E. histolytica* could stimulate proinflammatory responses in wild-type (WT) high MUC2 mucin-producing goblet-like cells and in clustered regularly interspaced palindromic repeats and CRISPR-associated protein 9 (CRISPR-Cas9) gene-edited *MUC2KO* cells was investigated. In response to live *E. histolytica* and soluble *E. histolytica* proteins, WT, and to a lesser extent, *MUC2KO* cells produced high levels of CXCL8. *Entamoeba histolytica* temporally induced greater levels of CXCL8 mRNA expression and protein secretion in WT versus *MUC2KO* cells, which was abrogated with alleviation of endoplasmic reticulum stress with the NADPH-oxidase inhibitor diphenyleneiodonium chloride. WT cells produced elevated reactive oxygen species that induced longer half-lives of CXCL8 transcripts, which was abrogated with diphenyleneiodonium chloride. Western blot and proteomic analyses revealed that WT cells, but not *MUC2KO* cells, were basally primed to respond to external stressors and responded to *E. histolytica* through rapid activation of the mitogen-activated protein kinase/extracellular signal-regulated kinase, mitogen-activated protein kinase/p38, and phosphatidylinositol 3-kinase/Akt pathways, to induce CXCL8. These results suggest that colonic goblet-like cells defend against *E. histolytica* infections by hypersecreting mucus and produce the chemokine, CXCL8, to recruit neutrophils.

## Graphical abstract

**Figure:**
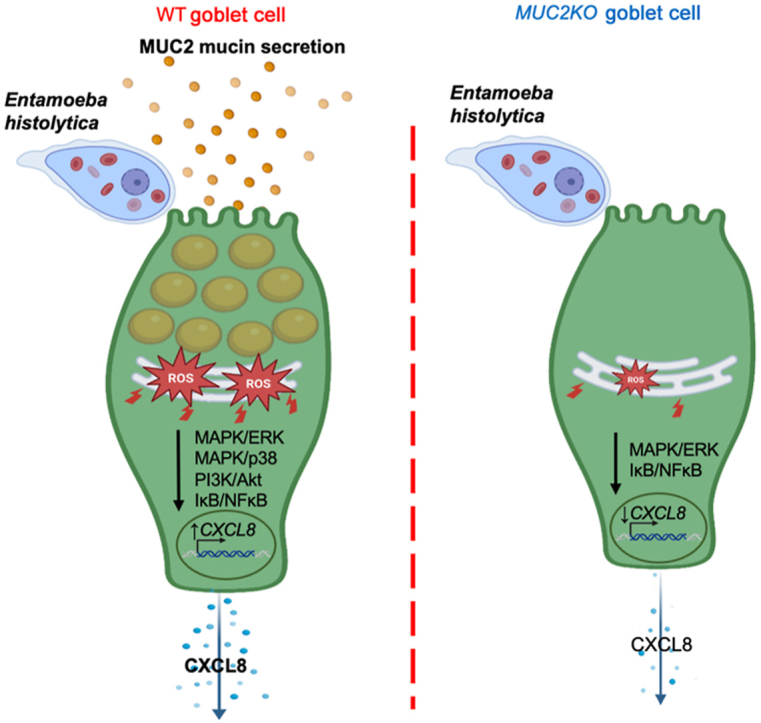

A thick mucus barrier protects the gastrointestinal tract from potential pathogens and toxins from the external environment.^1^ This essential barrier is produced by specialized secretory epithelial cells called goblet cells. In the colon, the primary component of mucus is mucin-2 (MUC2), a high-molecular-weight glycoprotein, which lubricates and protects the single layer of epithelial cells.^1^ This mucus barrier is organized in two layers: the inner dense layer is sterile and in direct contact with epithelial cells and an outer loose layer that serves to house commensal microbiota.^2^ The production of mucus is metabolically stressful as the MUC2 protein undergoes several steps of glycosylation before it is secreted into the lumen. The MUC2 apoprotein first undergoes *N*-glycosylation at the endoplasmic reticulum (ER) to form dimers,^3^ followed by extensive *O*-glycosylation at the Golgi apparatus, which gives it its characteristic water-retentive features. The most abundant *O*-glycans in MUC2 are galactose, *N*-acetyl galactosamine, and *N*-acetyl glucosamine.^4^

*Entamoeba histolytica* is a human intestinal protozoan parasite that is the causative agent of amebiasis or amebic dysentery, and a leading cause of diarrheal diseases worldwide, especially in less industrialized countries.^5^ Following ingestion of fecal-contaminated food or water containing infective cysts, excystation occurs in the lower ileum and colon. This produces motile trophozoites that use their surface galactose and *N*-acetyl D-galactosamine lectins to bind to colonic mucins during colonization. Encystation of *E. histolytica* occurs in the colon, and cysts are excreted in stool to continue its fecal-oral life cycle.^5^ Approximately 90% of infections are asymptomatic, with *E. histolytica* remaining in the lumen of the colon. However, invasive infections can result in cramping, abdominal pain, watery or bloody diarrhea, and weight loss, with colitis characterized by flask-shaped ulceration and inflammation characterized by rapid recruitment of neutrophils.^6^ Columnar epithelial cells produce NF-κβ–regulated proinflammatory cytokines in response to amebic infection, including IL-1β and CXCL8 (alias IL-8).^7^ Previous *in vitro* studies have revealed that CXCL8 is induced from colonic epithelial cells without direct *E. histolytica*–enterocyte contact.^8^ CXCL8 production is regulated, in part, by the activation of prostaglandin E_2_ receptor 4 (EP4) receptors on colonic epithelial cells via the synthesis of *E. histolytica*–derived prostaglandin E2.^9^ CXCL8 is a potent chemokine that functions to recruit neutrophils to sites of inflammation and results in neutrophil degranulation and respiratory burst.^10^ The 72–amino acid biologically active form of CXCL8 is secreted from cells once the signal peptide is removed from the 99–amino acid precursor form.^10^ The promoter region for CXCL8 includes binding sites for NF-κβ, activating protein-1, and cytosine-adenosine-adenosine-thymidine/enhancing binding protein (CAAT/EBP).^11^ CXCL8 participates in other functions, such as angiogenesis, tumor progression, mitosis, and tissue remodeling.^11^

Hypersecretion and depletion of mucus from goblet cells is characteristic of acute *E. histolytica* infections. Whether goblet cells depleted of MUC2 mucin stores can produce other proteins in innate host defense against *E. histolytica* is not known. In this study, whether secretion of proinflammatory cytokines by goblet-like cells was influenced by the biosynthesis and production of MUC2 in response to *E. histolytica* was investigated. MUC2 biosynthesis in goblet-like cells resulted in ER stress-driven reactive oxygen species (ROS) production that stabilized CXCL8 transcripts and produced high levels of CXCL8 protein in response to *E. histolytica*. Mechanistically, *E. histolytica* activated multiple proinflammatory signaling pathways in wild-type (WT) but not in *MUC2KO* cells, including mitogen-activated protein kinase (MAPK)/extracellular signal-regulated kinase (ERK), MAPK/p38, and phosphatidylinositol 3-kinase (PI3K)/Akt. These results uncover a previously uncharacterized role of colonic goblet-like cells in the production of chemokines as innate host defense against *E. histolytica*.

## Materials and Methods

### Human Goblet-Like Cells

The human colon cancer cell line LS174T (ATCC, Manassas, VA; CL-188) was maintained in complete Eagle's minimum essential medium (Lonza, Basel, Switzerland), 10% heat-inactivated fetal calf serum (Millipore Sigma, Burlington, MA), 1 mmol/L L-glutamine (Sigma-Aldrich, St. Louis, MO), 10 mmol/L HEPES, and penicillin-streptomycin (100 U/mL; 100 μg/mL; Gibco, Waltham, MA). Cells were maintained in a humidified incubator with 5% CO_2_. Medium was changed every 2 days, and cells were passed once a week, as the cells reached 80% confluency, using a trypsin solution. For protein and RNA extraction, LS174T cells were plated onto 12-well plates (1.0 × 10^5^ cells/well) in 2 mL of complete Eagle's minimum essential medium. Experiments were performed using approximately 80% confluent cells, and fresh medium was added to the culture 24 hours before each experiment. The clustered regularly interspaced palindromic repeats and CRISPR-associated protein 9 (CRISPR-Cas9) system was previously used to gene edit MUC2 in LS174T cells, as previously described.^12^ In brief, the *MUC2KO* cell line was previously generated by transfecting LS174T cells with a plasmid containing MUC2-specific guided-RNA sequences and selectively cloned with 4.24 μmol/L puromycin. Successful deletion of the MUC2 gene was confirmed by PCR, Western blot analysis, and confocal microscopy after 3 weeks.^12^

### Cultivating and Harvesting *E. histolytica*

The virulent *E. histolytica* strain, HM1:IMSS, was grown in axenic conditions in TYI-S-33 medium with 100 U/mL penicillin and 6.86 μmol/L streptomycin sulfate at 37°C in sealed borosilicate glass tubes. To maintain high virulence, trophozoites were regularly passaged through gerbil livers. Amebae were harvested after 72 hours during their logarithmic growth phase by chilling tubes on ice for 5 minutes and washing with cold phosphate-buffered saline. Amebae were then centrifuged at 200 × *g* for 5 minutes at 4°C. Viable amebic trophozoites were determined by the trypan blue exclusion assay. Soluble amebic protein (SAP) was prepared by lysing ameba with three consecutive freeze-thaw cycles at –80°C. Amebae were then centrifuged at 14,000 × *g* for 10 minutes at 4°C, and the clear supernatant was collected. Protein concentration was determined using the Pierce bicinchoninic acid (BCA) protein assay kit (Thermo Fisher Scientific, Mississauga, ON, Canada), according to the manufacturer's protocol.

### Quantitative Real-Time PCR

Total RNA was extracted from cells using the E. Z.N.A Total RNA kit (Omega Bio-TEK, Norcross, GA), as per the manufacturer's instruction. The yield and purity of the RNA were measured using a NanoDrop 1000 spectrophotometer (Thermo Fisher Scientific) at 260/280 nm. A total of 500 ng of RNA was reverse transcribed using the qScript cDNA synthesis kit (Quantabio, Beverly, MA). Real-time quantitative PCR was performed with each reaction mixture containing 1:10 cDNA dilutions, SYBR Green PCR Master Mix (Quantabio), and 1 μmol/L primers (forward + reverse). Reactions were done on a Rotor-Gene 3000 real-time quantitative PCR system (Qiagen, Toronto, ON, Canada) in duplicate, and results were analyzed using the 2^−ΔΔC_T_^ method and expressed as fold changes relative to the housekeeping gene, glyceraldehyde-3-phosphate dehydrogenase (GAPDH). The following primer sequences were used: GAPDH, 5′-TGATGACATCAAGAAGGTGGTGAAG-3′ (forward) and 5′-TCCTTGGAGGCCATGTGGGCCAT-3′ (reverse); MUC2, 5′-CCTGGCCCTGTCTTTGG-3′ (forward) and 5′-CTTCAGGTGCACAGCAAATTC-3′ (reverse); and CXCL-8, 5′-CTGGCCGTGGCTCTCTTG-3′ (forward) and 5′-CCTTGGCAAAACTGCACCTT-3′ (reverse).

### mRNA Stability

Cells were stimulated with phorbol 12-myristate 13-acetate (PMA) for 2 hours or live *E. histolytica* or SAP for 1 hour to induce maximal transcriptional responses before being treated with actinomycin D (7.97 μmol/L) to inhibit transcription. mRNA was taken at 0, 0.5, 1, 2, 4, 6, 8, 12, and 24 hours after actinomycin D treatment. A total of three to four individual samples were taken at each time point from three independent experiments (*n* = 9 to 12). mRNA was quantified through quantitative RT-PCR, and linear regression analysis was used to calculate mRNA half-lives. For analysis, duplicate cycle threshold values were averaged, the average time = 0 values were subtracted, and then the fold change was calculated. Half-lives of transcripts were determined by linear regression analysis. Translation was inhibited with treatment of cycloheximide (10 μg/mL) following stimulation with PMA, live *E. histolytica*, or SAP, and analyzed the same way.

### SDS-PAGE Western Blot Analysis

Cells were washed with cold 1× phosphate-buffered saline and resuspended in 300 μL complete protein lysis buffer (20 mmol/L Tris-HCl, pH 7.5, 100 mmol/L NaCl, 0.5% Triton X-100, 1 mmol/L EDTA, 0.1% SDS, phenylmethylsulfonyl fluoride, and a protease inhibitor cocktail; Sigma-Aldrich), before being sonicated. Lysis buffer contained sodium orthovanadate and sodium fluoride when visualizing phosphorylated proteins. Samples were then left on ice for 30 minutes before being centrifuged at 8000 × *g* for 10 minutes to remove the pelleted debris. The remaining protein was quantified by a Thermo Scientific Pierce bicinchoninic acid protein assay kit, according to the manufacturer's protocol, adjusted to equal concentrations, resuspended in Laemmli buffer, and boiled for 5 minutes. Samples were resolved on 7.5%, 10%, 12%, or 15% SDS-polyacrylamide gels before being transferred onto a nitrocellulose membrane. The membranes were blocked with 5% milk protein in phosphate-buffered saline and 0.1% Tween or 3% bovine serum albumin (for phosphorylated protein detection) at room temperature before incubating with primary antibodies at 4°C overnight. As a housekeeping marker, mouse monoclonal GAPDH (Millipore Sigma) was used as a loading control. Corresponding horseradish peroxidase–conjugated secondary antibodies were used in 3% milk powder in phosphate-buffered saline and 0.1% Tween (or 3% bovine serum albumin for phosphorylated proteins), then blots were visualized using the Immobilon Western Chemiluminescent HRP Substrate detection reagent (EMD Millipore, Burlington, MA). Blots were detected on the Chemidoc MP Imaging System (Bio-Rad, Hercules, CA), and images were quantified and analyzed using Image Lab software version 6.0 (Bio-Rad). Primary antibodies used were as follows: anti-GAPDH (Millipore Sigma; 1:10,000), anti–activating transcription factor (ATF) 4 (Cell Signaling Technology, Danvers, MA; number 11815; 1:1000), anti-binding immunoglobulin protein (BiP) (Cell Signaling Technology; number 3177; 1:4000), anti-ATF6 (Cell Signaling Technology; number 65880; 1:1000), anti–phosphorylated p44/42 MAPK (ERK1/2; Cell Signaling Technology; number 9101; 1:1000), anti-p44/42 (ERK1/2; Cell Signaling Technology; number 9102; 1:1000), anti–phosphorylated p38 MAPK (Cell Signaling Technology; number 9211; 1:1000), anti-p38 α/β MAPK (Santa Cruz Biotechnology, Dallas, TX; sc-7972; 1:1000), anti–phosphorylated c-Jun N-terminal kinase (JNK) (Santa Cruz Biotechnology; sc-6254; 1:1000), anti-JNK (Santa Cruz Biotechnology; sc-7345; 1:1000), anti–phosphorylated PI3K (Ser473; Cell Signaling Technology; number 4051; 1:800), anti-PI3K (Cell Signaling Technology; number 9272; 1:1000), anti–phosphorylated IκBα (s32; Cell Signaling Technology; number 28595; 1:1000), anti-IκBα (Cell Signaling Technology; number 4812; 1:1000), anti–phosphorylated NF-κβ p65 (Santa Cruz Biotechnology; sc-101752; 1:1000), and anti–NF-κβ p65 (Santa Cruz Biotechnology; sc-8008; 1:1000).

### Cytotoxicity (LDH) Assay

Lactate dehydrogenase (LDH) in cell culture media was measured using the Promega CytoTox-ONE homogeneous membrane integrity assay (G7890; Fisher Scientific) following the manufacturer's instructions. Percentage cell death was calculated using the equation: (LDH reading – background)/(maximal LDH release – background) × 100%, and relative cell death to control was calculated by subtracting % LDH release from non-stimulated cells.

### ROS Activity Assay

A 2′,7′-dichlorofluorescin diacetate (DCFDA) cellular ROS assay kit (Abcam, Cambridge, UK; ab113851) was used to measure hydroxyl, peroxyl, and other ROS production within LS174T WT and *MUC2KO* cells, per manufacturer's instructions. In brief, cells were seeded in 96-well plates and stained with DCFDA, and fluorescence was measured with excitation/emission at 485/535 nm after 4 hours. Relative ROS production was calculated by comparing fluorescence fold change compared with untreated LS174T WT cells.

### Cytokine Secretion

Production and secretion of proinflammatory cytokines in the cell culture supernatant was assessed using a Human Cytokine Proinflammatory Focused 15-plex Discovery Assay Array Luminex multiplex assay (Eve Technologies, Calgary, AB, Canada). Cells were treated for 1 hour with various stimuli, and the supernatants were collected. Untreated cell supernatants were used for controls. Supernatants were spun down to remove any debris. CXCL8 in cell supernatants was measured using a human IL-8/CXCL8 DuoSet enzyme-linked immunosorbent assay kit, according to manufacturer's instructions (R&D Systems, Minneapolis, MN). In short, high binding clear polystyrene enzyme-linked immunosorbent assay strip plates were coated with the capture antibody in phosphate-buffered saline overnight at room temperature. Wells were blocked for a minimum of 1 hour the next day, then samples and standards were incubated for at least 2 hours, followed by detection antibody incubation for 2 hours. Streptavidin–horseradish peroxidase was added for 20 minutes, followed by substrate solution for 20 minutes in the dark. Reactions were stopped by adding stop solution, and the OD of each well was determined immediately using a microplate reader set to 450 nm, with wavelength correction set to 570 nm. Wells were aspirated and washed three times between all steps, except when adding the stop solution, and incubations were done at room temperature. Samples were diluted accordingly with reagent diluent to ensure readings were within the assay range of 31.2 and 2000 pg/mL.

### Proteomic Analysis

All mass spectrometry (MS) experiments were performed by the Southern Alberta Mass Spectrometry core facility at the University of Calgary, Canada. Control and *E. histolytica* (SAP) stimulate WT and *MUC2KO* cells were used for shotgun proteomic analysis, as previously described.^13^ In brief, samples were lysed [1% SDS, 0.1 mol/L EDTA in 200 nmol/L HEPES (pH 8), protease inhibitor tablets (Roche, Basel, Switzerland)] and denatured (10 mmol/L dithiothreitol) before being alkylated by incubation with 15 mmol/L iodoacetamide in the dark for 25 minutes at room temperature. Samples were then incubated for 18 hours at 37°C with isotopically heavy [40 mmol/L formaldehyde-13C, d_2_ solution (13CD2O) + 20 mmol/L NaBH3CN (sodium cyanoborohydride)] or light labels [40 mmol/L light formaldehyde (CH2O) + 20 mmol/L NaBH3CN] to label peptide α- and ε-amines. Samples were then subjected to C18 chromatography before being subjected to liquid chromatography and tandem mass spectrometry. Analysis was performed on an Orbitrap Fusion Lumos Tribrid MS (Thermo Scientific) operated with Xcalibur version 4.0.21.10 and coupled to a Thermo system. Tryptic peptides (2 mg) were loaded onto a C18 trap (75 mm × 2 cm; Acclaim PepMap 100, part number 164946; Thermo Scientific) at a flow rate of 2 mL/minute of solvent A (0.1% formic acid and 3% acetonitrile in liquid chromatography–MS grade water). Peptides were eluted using a 120-minute gradient from 5% to 40% (5% to 28% in 105 minutes, followed by an increase to 40% B in 15 minutes) of solvent B (0.1% formic acid in 80% liquid chromatography–MS grade acetonitrile) at a flow rate of 0.3 mL/minute and separated on a C18 analytical column (75 mm × 50 cm; PepMapRSLC C18; part number ES803; Thermo Scientific). Peptides were then electrosprayed using 2.3-kV voltage into the ion transfer tube (300°C) of the Orbitrap Lumos operating in positive mode. The Orbitrap first performed a full MS scan at a resolution of 120,000 full width at half-maximum (FWHM) to detect precursor ions with an m/z between 375 and 1575 and a 2 to 7 charge. The Orbitrap Auto Gain Control and the maximum injection time were set at 4 × 105 and 50 milliseconds, respectively. The Orbitrap was operated using the top speed mode with a 3-second cycle time for precursor selection. The most intense precursor ions presenting a peptidic isotopic profile and having an intensity threshold of at least 5000 were isolated using the quadrupole and fragmented with higher-energy collision dissociation (30% collision energy) in the ion routing multipole. The fragment ions (MS2) were analyzed in the ion trap at a rapid scan rate. The Auto Gain Control and the maximum injection time were set at 1 × 104 and 35 milliseconds, respectively, for the ion trap. Dynamic exclusion was enabled for 45 seconds to avoid the acquisition of the same precursor ion with a similar m/z (±10 parts per million). Spectral data were matched to peptide sequences in the human UniProt protein database using Mascot (*https://www.matrixscience.com*, last accessed February 8, 2023). The cleavage site specificity was set to trypsin/P for the proteomics data, with up to two missed cleavages allowed. After protein identification, the resulting hits were submitted to Metascape (20) for bioinformatic analysis to generate the Gene Ontology pathway analyses and transcription factor analysis presented. MaxQuant software version 2.6.7.0 (Max Planck Institute of Biochemistry, Munich, Germany, *https://www.maxquant.org*) was used for a peptide-spectrum match at a 1% false discovery rate, and log2 of values were used to interpret changes in protein abundance. Venn diagrams highlighting common interesting proteins involved in pathways of interest were generated with Venny 2.0 (*https://bioinfogp.cnb.csic.es/tools/venny*).

### Statistical Analysis

All experiments shown are representative of at least three independent experiments unless otherwise stated. All statistical analyses were conducted using GraphPad Prism 9 (GraphPad Software, San Diego, CA). Groups with different treatments were analyzed with two-way analysis of variance with the Tukey *post hoc* test. The *t*-test was used when only two groups were compared. Slopes of mRNA half-lives were calculated by linear regression and compared by analysis of covariance. Significance was assumed at *P* < 0.05 (∗*P* < 0.05, ∗∗*P* < 0.01, ∗∗∗*P* < 0.001, and ∗∗∗∗*P* < 0.0001). Results presented in histograms are displayed as means with error bars depicting ±SD. To control for potential off-target effects of CRISPR-Cas9 gene editing, all statistical analyses were conducted comparing each cell line with its own unstimulated controls.

## Results

### MUC2 Mucin Biosynthesis Induces ER Stress in Colonic Goblet-Like Cells Driven by Reactive Oxygen Species

**Figure 1:**
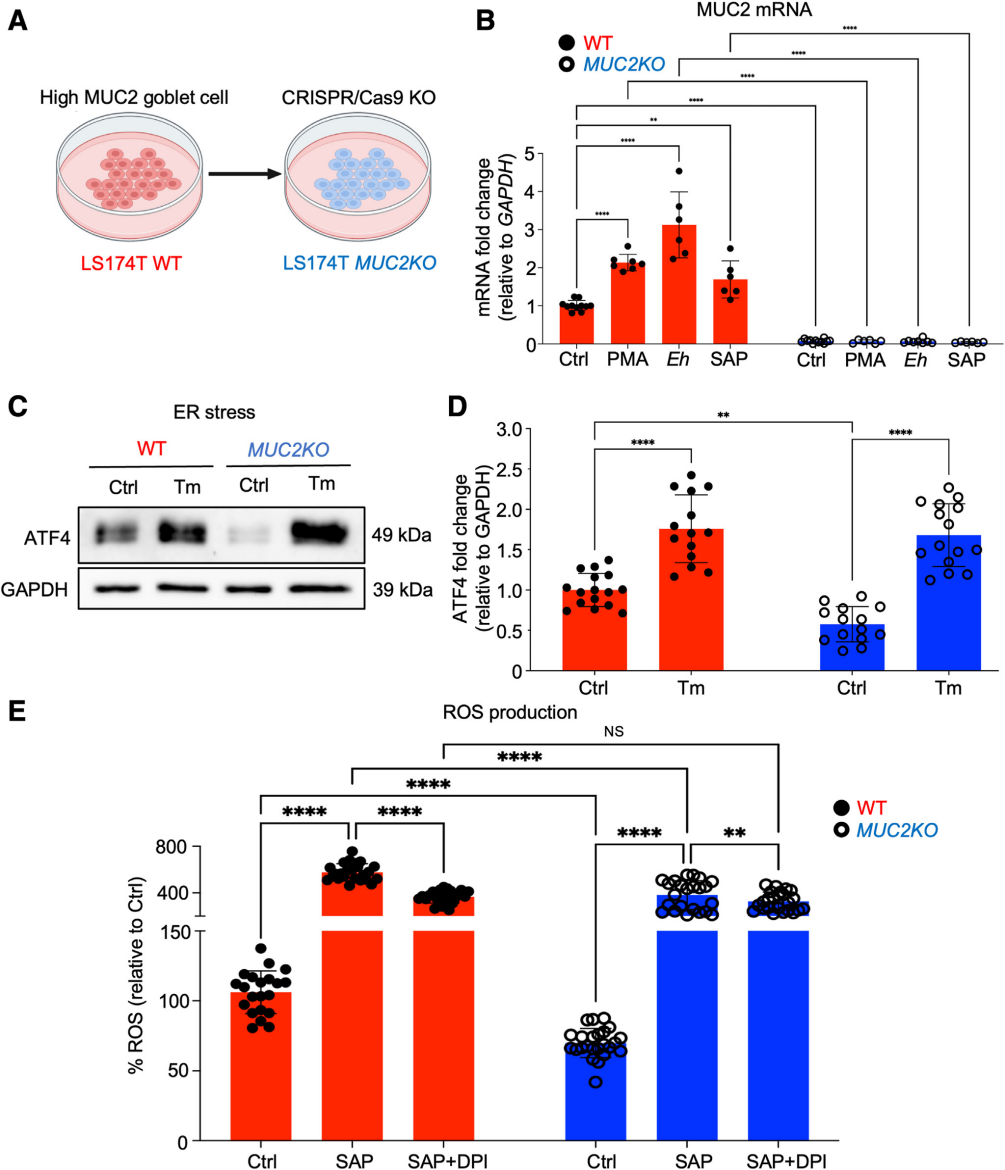
Mucin production induces endoplasmic reticulum (ER) stress in colonic goblet-like cells driven by high reactive oxygen species (ROS) production. **A:** Schematic of goblet cell line, LS174T wild type (WT), and CRISPR/Cas9 *MUC2KO*. Figure was generated with BioRender.com (Toronto, ON, Canada). **B:***MUC2* mRNA expression in WT and CRISPR/Cas9 *MUC2KO* in response to mucus secretagogues. Phorbol 12-myristate 13-acetate (PMA) treatment at 1 μmol/L, 2 hours. *Entamoeba histolytica* (*Eh*) treatment at 40,000 trophozoites per well, 2 hours. Soluble amebic protein (SAP) treatment was at 100 μg/mL, 2 hours. **C:** The ER stress marker, activating transcription factor 4 (ATF4), was measured by Western blot analysis in WT and *MUC2KO* cells basally. Tunicamycin (Tm; 10 μg/mL; 18 hours) was used as a positive control (Ctrl). **D:** Densitometry analysis of ATF4 expression in WT and *MUC2KO* cells basally and in response to tunicamycin treatment. **E:** ROS production was measured by a 2′,7′-dichlorofluorescin diacetate assay in WT and *MUC2KO* cells. SAP treatment was at 100 μg/mL, 1 hour, and diphenyleneiodonium chloride (DPI), an NAPDH-oxidase inhibitor, was used as a pretreatment at 10 μmol/L, 30 minutes. ∗∗*P* < 0.01, ∗∗∗∗*P* < 0.0001. GAPDH, glyceraldehyde-3-phosphate dehydrogenase; KO, knockout; NS, not significant.

To determine whether ER stress induced by high MUC2 mucin biosynthesis can affect the cells' ability to produce proinflammatory cytokines, WT and a CRISPR-Cas9 gene-edited *MUC2KO* LS174T goblet-like cell lines were used (Figure 1A^12^). WT cells secrete MUC2 mucin constitutively and in response to mucus secretagogues.^14^ To ensure that these cells could be used to recapitulate typical goblet cell biology, WT and *MUC2KO* cells were treated with mucus secretagogues, PMA, *E. histolytica,* or SAPs. *MUC2* gene expression was significantly up-regulated in WT, but not in *MUC2KO* cells (Figure 1B). ATF4 is a transcription factor that controls a wide range of genes to regulate cellular stress responses, and is also an ER stress marker. To determine whether mucin biosynthesis was driving ER stress, ATF4 expression was measured by Western blot (Figure 1, C and D). Under basal conditions at which MUC2 is constitutively produced, ATF4 expression was significantly higher in WT compared with that in *MUC2KO* cells, indicating that mucin production induces ER stress at steady states (Figure 1, C and D). The positive control, tunicamycin, an *N*-glycosylation inhibitor, led to an accumulation of misfolded proteins in the ER that induced stress.^15^ This corresponded to significantly higher expression of ATF4 in both cell lines, and confirmed that genetic deletion of *MUC2* had no effect on the cell's ability to mount a stress response (Figure 1, C and D). Taken together, these findings indicate that the increased expression of ER stress marker, ATF4, in mucin-producing WT goblet-like cells at steady states was due to ER stress driven by the biosynthesis and exocytosis of MUC2 mucin.

To determine whether MUC2 biosynthesis was driving the production of ROS, ROS was measured with the DCFDA assay in cells basally and in response to SAP stimulation. SAP was used in this experiment to prevent live *E. histolytica*–induced cell death. Briefly, lysed *E. histolytica* components allowed the effects of *E. histolytica* proteins in contact with goblet cells in the absence of cell death caused by live *E. histolytica* to be interrogated. At basal states, WT cells produced significantly more ROS compared with *MUC2KO* cells (Figure 1E). In response to SAP, ROS production was significantly increased in both cell lines, but was significantly higher in WT cells (Figure 1E). ROS production in response to SAP was significantly inhibited with the addition of an NAPDH-oxidase inhibitor, diphenyleneiodonium chloride (DPI), in both cell lines (Figure 1E). These results reveal that MUC2 mucin production induced ROS production constitutively and could also be induced by *E. histolytica* in goblet-like cells, which may play a role in further provoking ER stress in these cells.

### *E. histolytica* Induces CXCL8 Transcription from Colonic Goblet-Like Cells

**Figure 2:**
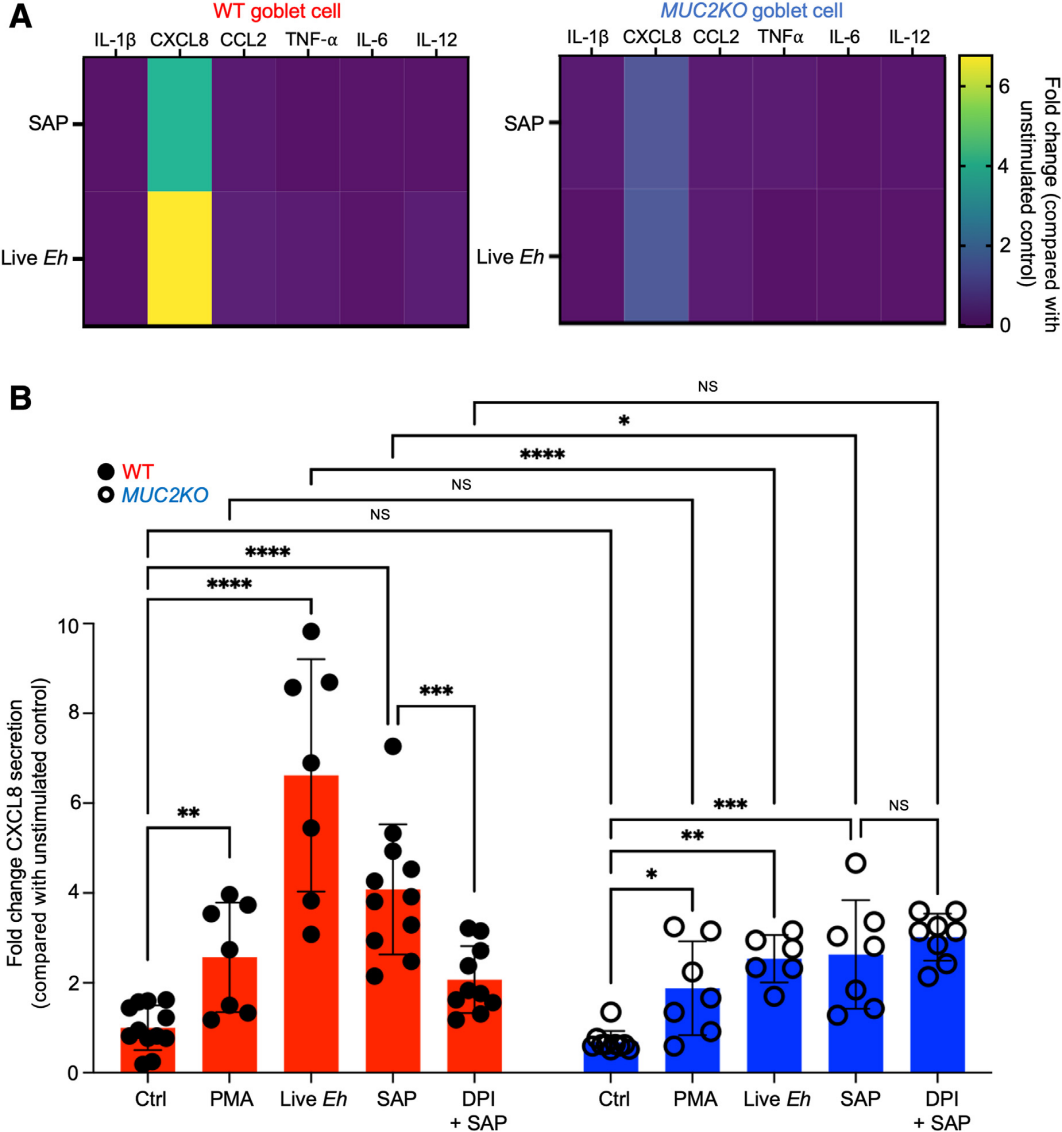
*Entamoeba histolytica* (*Eh*) induces CXCL8 secretion from colonic goblet-like cells. **A:** Proinflammatory cytokine release in the supernatants of LS174T wild-type (WT) and *MUC2KO* cells, as measured by a 15-plex Luminex assay. Soluble amebic protein (SAP) was used at 100 μg/mL, and live *E. histolytica* was used at 40,000 trophozoites per well for 1 hour. Negligible cytokine release was not shown. **B:** CXCL8 secretion measured in the supernatants of WT and *MUC2KO* cells was measured by a CXCL8-specific enzyme-linked immunosorbent assay after 1 hour of treatment. Phorbol 12-myristate 13-acetate (PMA) was used at 1 μmol/L, SAP at 100 μg/mL, live *E. histolytica* at 40,000 trophozoites per well, and diphenyleneiodonium chloride (DPI) pretreatment at 10 μmol/L for 30 minutes. ∗*P* < 0.05, ∗∗*P* < 0.01, ∗∗∗*P* < 0.001, and ∗∗∗∗*P* < 0.0001. CCL, chemokine (C-C motif) ligand; Ctrl, control; NS, not significant; TNF-α, tumor necrosis factor-α.

To determine whether WT and *MUC2KO* cells mounted differential proinflammatory cytokine responses, cells were treated with either live *E. histolytica* or SAP for 1 hour, and the supernatants were analyzed by a 15-plex Luminex assay. Six of the 15 cytokines measured were detected in response to live *E. histolytica* or SAP, but by far, the most dominant secreted chemokine measured was CXCL8 (Figure 2A). This was in line with previous *E. histolytica* studies that found CXCL8 to be one of the predominant chemokines observed in symptomatic *E. histolytica* infections.^16^ However, whether goblet cells contributed to the proinflammatory milieu in this context was not previously known. Apart from CXCL8, the proinflammatory response in *MUC2KO* cells was less pronounced regardless of the stimuli. On the basis of these findings, the secretion of CXCL8 was quantified by enzyme-linked immunosorbent assay in WT and *MUC2KO* cells in response to *E. histolytica* and PMA stimulation after 1-hour exposure. In response to PMA, which stimulates mucus production via activation of the protein kinase C pathway,^17^ both cell lines secreted significantly more CXCL8 compared with unstimulated controls. However, there were no significant differences in CXCL8 secretion between the two cell lines (Figure 2B). Treatment with live *E. histolytica* resulted in the most significant secretion of CXCL8 from both cell lines, with significantly more CXCL8 secretion from WT compared with *MUC2KO* cells (Figure 2B). SAP treatment similarly induced significant secretion of CXCL8 from both cell lines, again with significantly more chemokine secretion from WT compared with *MUC2KO* cells (Figure 2B). When ER stress was alleviated in WT cells pretreated with DPI to reduce ROS production, CXCL8 secretion was significantly reduced when compared with SAP treatment alone, comparable to CXCL8 secretion from *MUC2KO* cells (Figure 2B). In contrast, DPI pretreatment had no effect on CXCL8 secreted in response to SAP in *MUC2KO* cells (Figure 2B). These findings indicate that ER stress induced by MUC2 mucin biosynthesis regulated chemokine responses. WT cells secreted significantly more CXCL8 in response to amebic challenges, and low-stressed *MUC2KO* cells are not biochemically defective in producing CXCL8.

**Figure 3:**
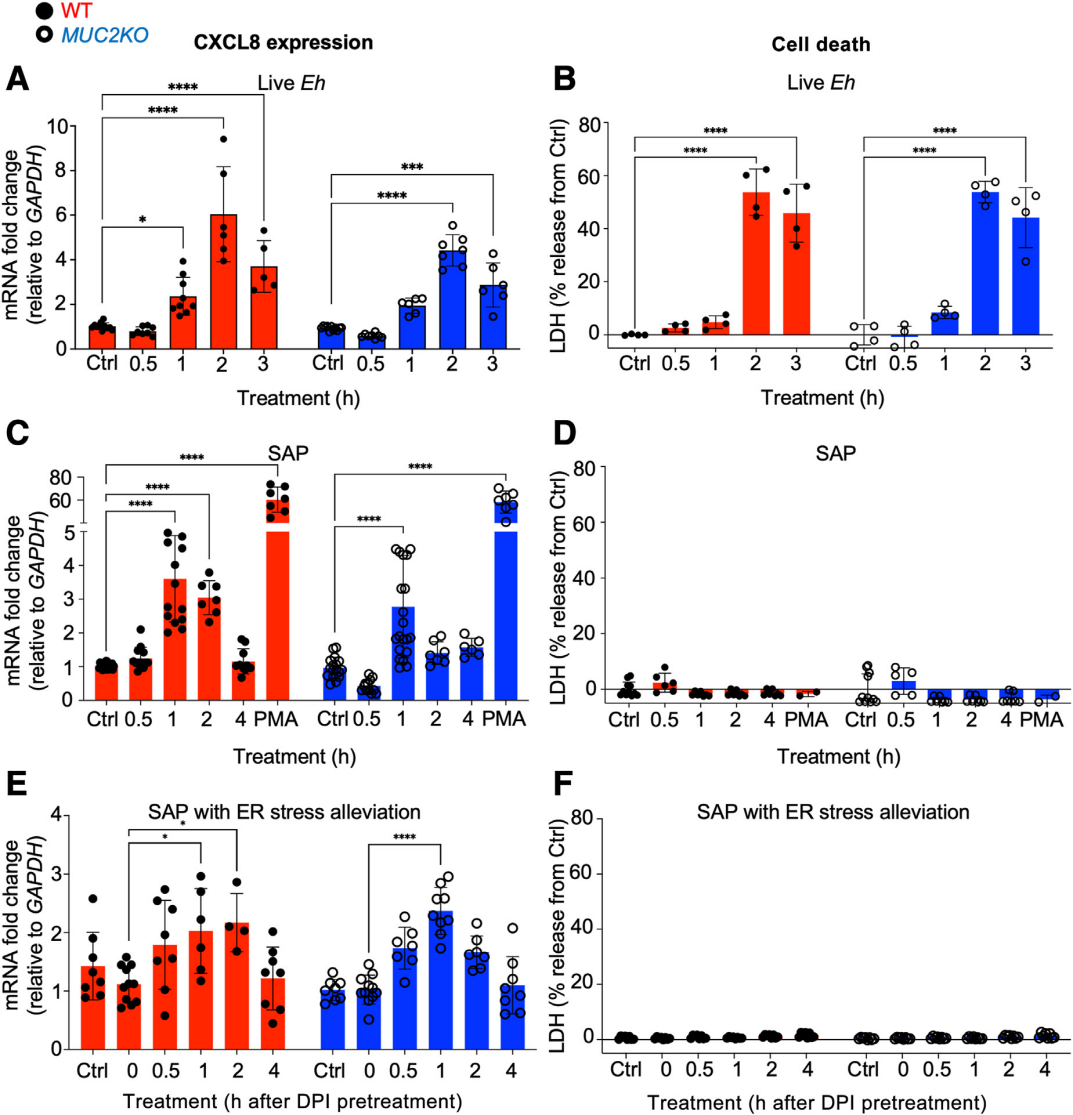
*Entamoeba histolytica* (*Eh*) induces CXCL8 transcription from colonic goblet-like cells, with or without cell death. **A**, **C**, and **E:** CXCL8 mRNA expression levels in wild-type (WT) and *MUC2KO* cells were measured by real-time quantitative PCR and compared with their corresponding untreated controls (Ctrls). Live *E. histolytica* was used at 40,000 trophozoites per well, soluble amebic protein (SAP) at 100 μg/mL, phorbol 12-myristate 13-acetate (PMA) at 1 μmol/L for 2 hours, and diphenyleneiodonium chloride (DPI) pretreatment at 10 μmol/L for 30 minutes. **B**, **D**, and **F:** Cell death was measured by a lactate dehydrogenase (LDH) assay in the supernatants of LS174T WT and *MUC2KO* cells and presented as percentage LDH release from their corresponding untreated controls. ∗*P* < 0.05, ∗∗∗*P* < 0.001, and ∗∗∗∗*P* < 0.0001. ER, endoplasmic reticulum.

To determine whether there were differential kinetics in the induction of CXCL8 in WT and *MUC2KO* cells, the transcription of CXCL8 in response to *E. histolytica* stimulation was followed temporally. In response to live *E. histolytica*, the expression of CXCL8 mRNA in both cell lines followed a bell-shaped curve, with peak transcription at 2 hours after treatment that declined thereafter (Figure 3A). In both cell lines, cell death measured by LDH was approximately 55% after 2-hour stimulation (Figure 3B). With SAP stimulation, CXCL8 mRNA transcripts peaked after 1 hour and decreased steadily thereafter in the absence of cell death (Figure 3, C and D). PMA treatment for 2 hours as a positive control maximally stimulated CXCL8 transcription in the absence of cell death in both cell lines (Figure 3C). When ER stress was alleviated with DPI pretreatment and challenged with SAP, transcription of CXCL8 was markedly inhibited in the absence of cell death compared with SAP treatment alone (Figure 3, C, E, and F). These results reveal that CXCL8 can be similarly transcribed in both cell lines in response to *E. histolytica* and is dependent, in part, on the ER stress response in the cells, as alleviation of ER stress diminished CXCL8 transcription in WT cells.

### Multiple Proinflammatory Signaling Pathways Are Activated in High Mucin-Producing Goblet-Like Cells

**Figure 4:**
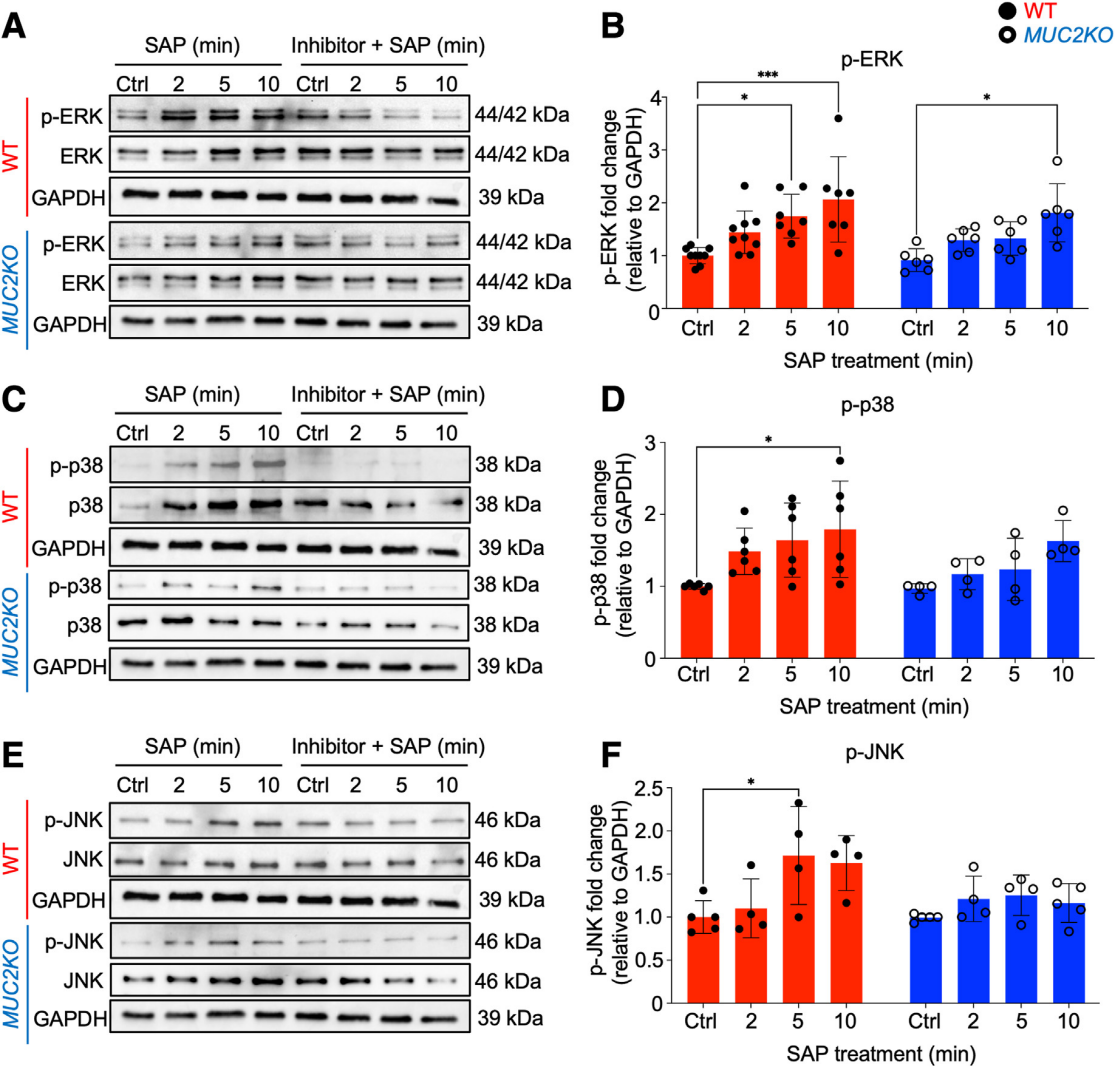
Soluble amebic protein (SAP) induces activation of classic proinflammatory mitogen-activated protein kinase (MAPK) signaling pathways in wild-type (WT) goblet-like cells. **A**, **C**, and **E:** Western blot analyses were probed to detect activation of MAPK/extracellular signal-regulated kinase (ERK), MAPK/p38, and MAPK/JNK pathways in response to SAP (100 μg/mL) in LS174T WT and *MUC2KO* cells, in the presence or absence of specific pathway inhibitors. The MAPK/ERK inhibitor, FR180204, was used at 10 μmol/L, the MAPK/p38 inhibitor, SB203580, was used at 10 μmol/L, and the MAPK/JNK inhibitor, SP600125, was used at 20 μmol/L. **B**, **D**, and **F:** Densitometry of Western blot analyses that were probed to detect activation of MAPK/ERK, MAPK/p38, and MAPK/JNK pathways in response to SAP (100 μg/mL) in LS174T WT and *MUC2KO* cells. Results are representative of at least three independent replicates. ∗*P* < 0.05, ∗∗∗*P* < 0.001. Ctrl, control; GAPDH, glyceraldehyde-3-phosphate dehydrogenase; p-ERK, phosphorylated ERK; p-JNK, phosphorylated JNK; p-p38, phosphorylated p38.

**Figure 5:**
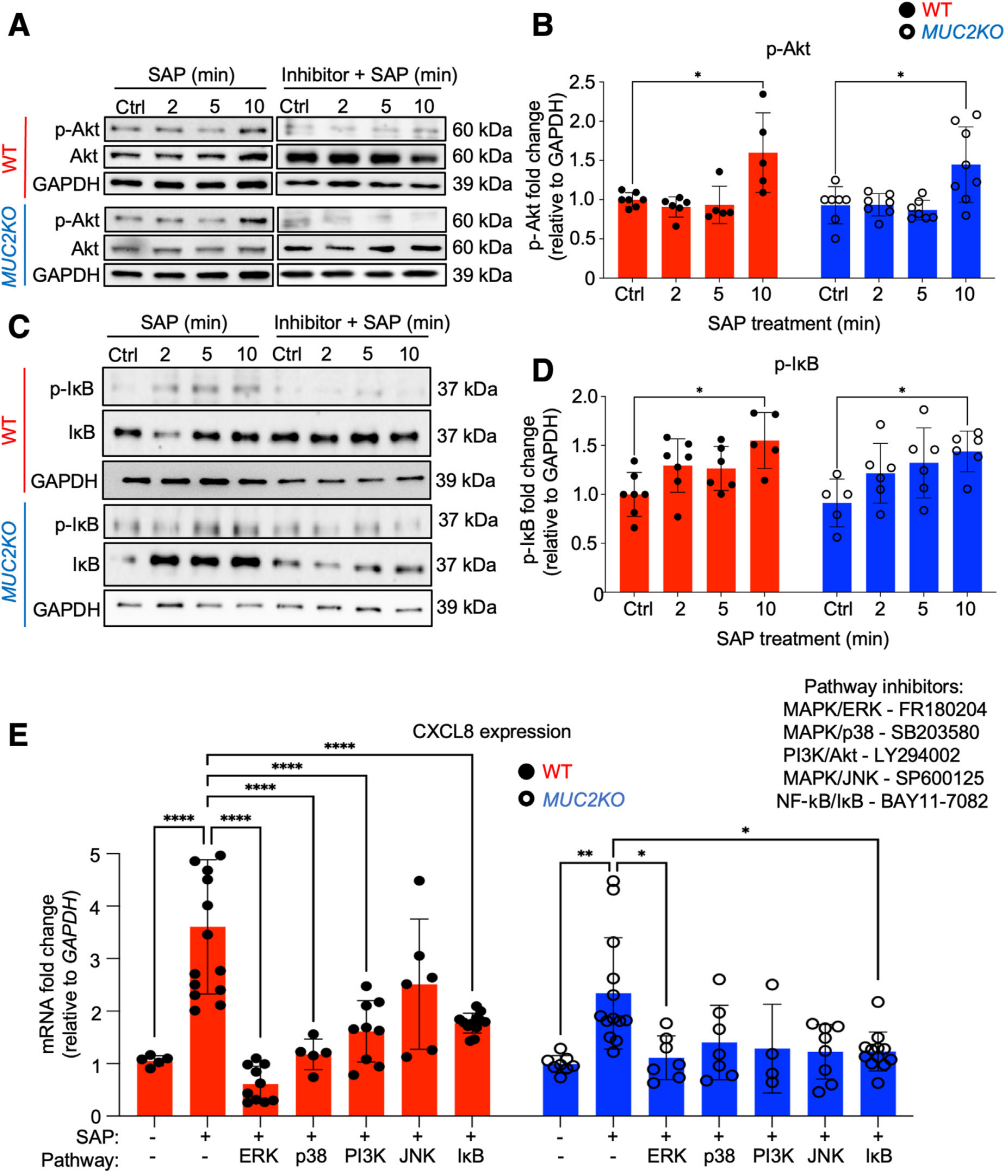
Multiple proinflammatory signaling pathways are involved in regulating CXCL8 expression in wild-type (WT) goblet-like cells. **A:** Western blot analyses were probed to detect activation of the phosphatidylinositol 3-kinase (PI3K)/Akt pathway in response to soluble amebic protein (SAP) in LS174T WT and *MUC2KO* cells. **B:** Densitometry of Western blot analyses that were probed to detect activation of the PI3K/Akt pathway in response to SAP in LS174T WT and *MUC2KO* cells. **C:** Western blot analyses were probed to detect activation of the NF-κβ/IκB pathway in response to SAP in LS174T WT and *MUC2KO* cells. **D:** Densitometry of Western blot analyses that were probed to detect activation of the NF-κβ/IκB pathway in response to SAP in LS174T WT and *MUC2KO* cells. **E:** CXCL8 mRNA expression, measured by real-time quantitative PCR in response to SAP (100 μg/mL) for 1 hour, and pretreated with various proinflammatory signaling pathway inhibitors. The mitogen-activated protein kinase (MAPK)/extracellular signal-regulated kinase (ERK) inhibitor, FR180204, was used at 10 μmol/L, the MAPK/p38 inhibitor, SB203580, was used at 10 μmol/L, the PI3K/Akt inhibitor, LY294002, was used at 50 μmol/L, the MAPK/JNK inhibitor, SP600125, was used at 20 μmol/L, and the NF-κB/IκB inhibitor, BAY11-7082, was used at 50 μmol/L. Results are representative of at least three independent replicates. ∗*P* < 0.05, ∗∗*P* < 0.01, and ∗∗∗∗*P* < 0.0001. Ctrl, control; GAPDH, glyceraldehyde-3-phosphate dehydrogenase; p-Akt, phosphorylated Akt; p-IκB, phosphorylated IκB.

The promoter region of CXCL8 includes various binding sites for the transcription factors NF-κB, activating protein-1, and CAAT/enhancing-binding protein.^11^ Therefore, the activation of proinflammatory signaling pathways that regulate CXCL8 from goblet-like cells in response to *E. histolytica* was explored by Western blot analysis. In WT cells, the MAPK pathways ERK1/2, p38, and JNK were significantly phosphorylated temporally in response to SAP treatment. Activation of these pathways was inhibited with the specific pathway inhibitors, FR180204, SB203580, and SP600125, respectively (Figure 4). ERK1/2, p38, and JNK were also phosphorylated in *MUC2KO* cells in response to SAP treatment, but the intensity of phosphorylation was minimal compared with that in WT cells (Figure 4). Only ERK1/2 was significantly phosphorylated in *MUC2KO* cells when measured by densitometry (Figure 4). In WT and *MUC2KO* cells, modest phosphorylation of Akt and IκBα were also observed by 10 minutes of SAP treatment (Figure 5, A–D). Phosphorylation of these proteins indicated that the major proinflammatory signaling pathways, MAPK/ERK, MAPK/p38, MAPK/JNK, PI3K/Akt, and NF-κβ/IκBα, were activated in goblet-like cells by *E. histolytica*. However, *MUC2KO* cells had diminished activation of these pathways.

To interrogate whether activation of these pathways affected CXCL8 transcription, WT and *MUC2KO* cells were treated with SAP for 1 hour (based on the results of Figure 3C) in the presence or absence of various specific proinflammatory pathway inhibitors (Figure 5E). In both cell lines, SAP-induced transcription of CXCL8 was significantly inhibited with the MAPK/ERK inhibitor, FR180204, and the NF-κβ/IκBα inhibitor, BAY11-7082, indicating that these pathways were conserved in goblet-like cells. No other pathway inhibitors reduced CXCL8 transcription in *MUC2KO* cells, whereas inhibition of the MAPK/p38 and PI3K/Akt pathways with SB203580 and LY294002, respectively, also significantly reduced CXCL8 transcription in WT cells (Figure 5E). These findings were unexpected as they reveal that *MUC2KO* cells unable to biosynthesize mucins that may have disrupted intracellular signaling pathways that regulate proinflammatory cytokines, perhaps because of the absence of endogenous ER stress (Figure 1C). Inhibiting the MAPK/p38, MAPK/JNK, or PI3K/Akt pathways did not significantly alter CXCL8 transcription in *MUC2KO* cells (Figure 5E), indicating that these pathways are not involved in regulating proinflammatory cytokine release from these cells in response to *E. histolytica.* Notably, inhibition of the MAPK/JNK pathway failed to reduce transcription of CXCL8 in WT cells (Figure 5E). This finding was intriguing as the MAPK/JNK signaling pathway activates the activating protein-1 transcription factor to promote transcripts of CXCL8 in other cells.^10^^,^^11^ Activation of the MAPK/p38 and PI3K/Akt signaling pathways was previously shown to stabilize proinflammatory cytokine mRNA transcripts, which could explain why significantly more CXCL8 was secreted from WT goblet-like cells^11^^,^^18^, ^19^, ^20^ (Figure 2A). Overall, these results revealed that although various proinflammatory signaling pathways could be activated by *E. histolytica*, the MAPK/ERK, MAPK/p38, PI3K/Akt, and NF-κβ/IκBα pathways specifically regulated the expression of CXCL8 in WT goblet-like cells, but the MAPK/p38 and PI3K/Akt signaling pathways were disrupted in *MUC2KO* cells.

### Proteins that Regulate Metabolism and Response to Stimuli Are Up-Regulated in WT Goblet-Like Cells Compared with *MUC2KO*

**Figure 6:**
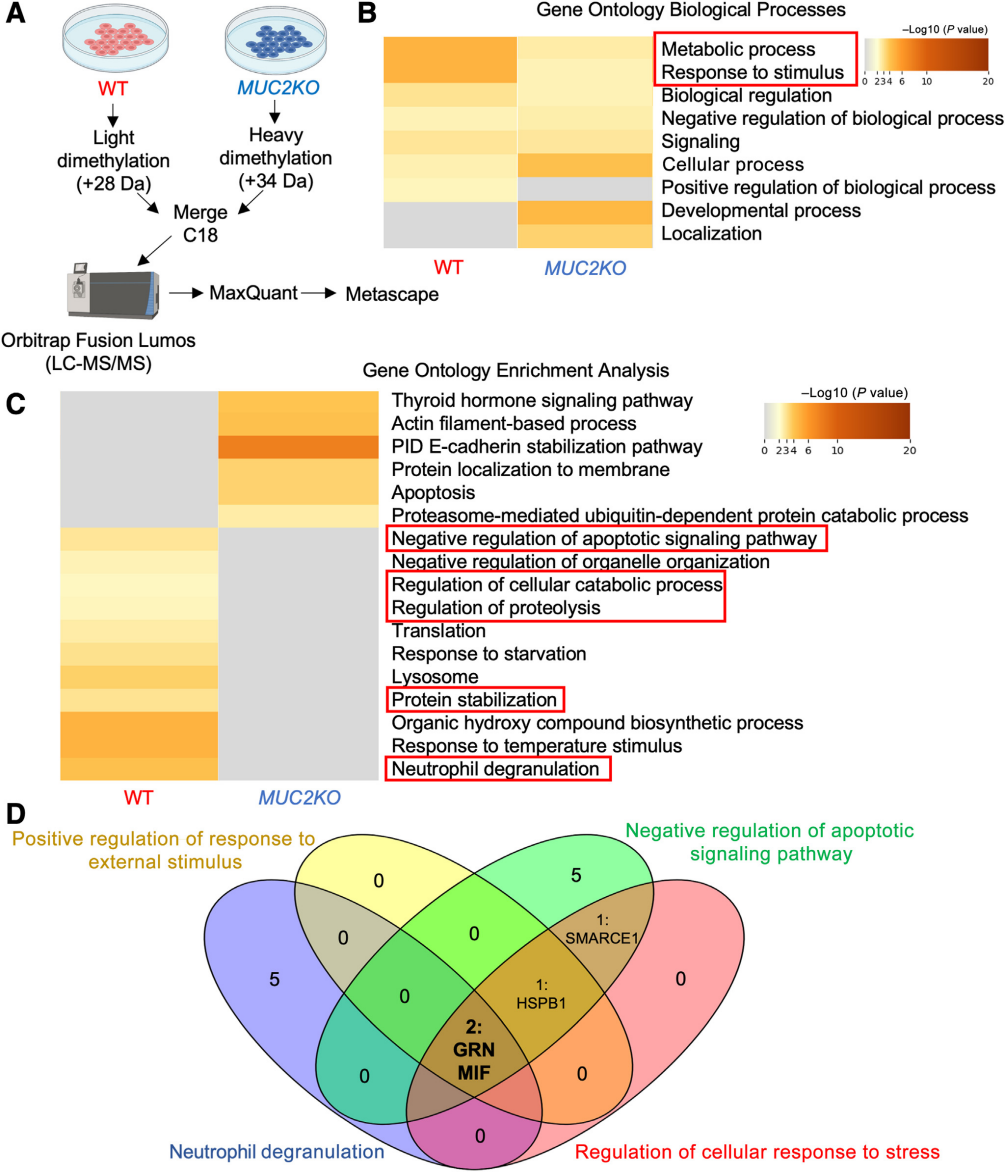
Wild-type (WT) goblet-like cells are enriched in CXCL8-regulatory proteins. **A:** Schematic of the sample preparation and workflow for shotgun proteomic analysis. Figure was generated with BioRender.com (Toronto, ON, Canada). **B:** Metascape analysis of the top enriched biological processes in WT and *MUC2KO* cells. Processes of interest are in **red boxed areas**. **C:** Metascape analysis showing Gene Ontology enrichment analysis of WT and *MUC2KO* cells. Pathways of interest are in **red boxed areas**. **D:** Venn diagram highlighting common interesting proteins involved in pathways of interest. Numbers refer to protein hits; proteins in pathways with overlapping sections listed out. Pathways of interests were determined by relevance to stress or immune responses. Venn diagram generated with Venny 2.0 (*https://bioinfogp.cnb.csic.es/tools/venny*). GRN, granulin; HSPB1, heat shock protein beta-1; LC-MS/MS, liquid chromatography–tandem mass spectrometry; MIF, macrophage migration-inhibitory factor; PID, pathway interaction database; SMARCE1, SWI/SNF-related maxtrix-associated actin-dependent regulator of chromatin subfamily E member 1.

To determine whether WT goblet-like cells were metabolically priming the cells to respond to stressors such as *E. histolytica*, and to uncover the pathways enriched, WT and *MUC2KO* cells were tagged with either heavy or light isotope labels and analyzed by shotgun proteomic analysis, followed by Metascape Gene Ontology enrichment analysis (Figure 6A and Supplemental Table S1). Following Gene Ontology enrichment analysis, pathways of interest were identified on the basis of their relevance to cellular stress or immune responses, and protein abundances were analyzed by log2 to determine statistical differences. Metascape analysis of the top enriched biological processes revealed that WT cells had increased activation of pathways involved in metabolic processes and responses to stimuli, compared with *MUC2KO* cells (Figure 6B). This was expected, as WT cells have high metabolic demands for amino acids and sugars to biosynthesize mucins. This was also in line with the higher stress proteins measured by Western blot analysis and ROS production measured by a DCFDA assay in WT cells compared with the *MUC2KO* cells (Figure 1, C–E). To uncover the specific gene pathways involved in these processes, Gene Ontology enrichment analysis from Metascape was conducted. *MUC2KO* cells were most enriched in proteins involved in the pathway interaction database (PID) E-cadherin stabilization pathway (Figure 6C). The PID E-cadherin stabilization pathway is involved in the stabilization and expansion of the E-cadherin adherens junction protein, which plays an important role during tumor metastasis, and is reduced in the presence of inflammatory cytokines in human epithelial cells.^21^^,^^22^ WT cells had more proteins involved in pathways involving negative regulation of apoptotic signaling, regulation of cellular catabolic process, regulation of proteolysis, protein stabilization, and neutrophil degranulation (Figure 6C). Enrichment of proteins involved in negative regulation of apoptotic signaling pathways, cellular catabolic processes, proteolysis, and protein stabilization reveals that WT cells may be able to sustain high levels of ER stress because of mucin production and when faced with a potential pathogen by activating the unfolded protein response to reduce the accumulation of misfolded proteins to alleviate ER stress.^23^ When the proteins involved in these pathways were compared, two proteins of interest, granulin (GRN) and macrophage migration-inhibitory factor (MIF), were identified (Figure 6D and Table 1). These two proteins were explored further as they have potential CXCL8-regulatory functions. GRN is a secreted cytokine-like molecule that stimulates CXCL8 secretion from bronchial epithelial cells.^24^^,^^25^ MIF, another cytokine, released from lymphocytes and fibroblasts, activates the MAPK signaling pathways to stimulate secretion of CXCL8 in inflammatory diseases.^26^, ^27^, ^28^ *E*. *histolytica* also encodes a homologue of MIF, which stimulates the secretion of CXCL8 from intestinal epithelial cells.^29^^,^^30^ These data suggest that GRN and MIF production from MUC2 mucin-producing goblet-like cells could potentially prime them to be more responsive to external stimuli by regulating CXCL8 expression.

**Table 1:** Attractive Protein Hits Identified in Pathways of Interest in WT and *MUC2KO* Cells at Basal States

Pathway	Gene symbol	Protein name	Log2 (KO:WT)
Neutrophil degranulation			–4.91236
	*CTSD*	Cathepsin D; cathepsin D light chain; cathepsin D heavy chain	–0.981280377549962
	*GNS*	N-acetylglucosamine-6-sulfatase	–0.833850176648613
	***GRN***	**Granulin**	**–0.928264784720231**
	***MIF***	**Macrophage migration inhibitory factor**	**–0.769452650396329**
	*PSMD2*	26S proteasome non-ATPase regulatory subunit 2	1.22780267550784
	*S100A11*	Protein S100-A11; protein S100-A11, N-terminally processed	0.947329519847386
	*PDXK*	Pyridoxal kinase	–1.19899038179497
Protein stabilization			–4.59602
	*ATP1B1*	Sodium/potassium-transporting ATPase subunit β-1	1.15898325116039
	*CTNND1*	Catenin δ-1	–0.807603808419224
	***GRN***	**Granulin**	**–0.928264784720231**
	*H1-5*	Histone H1.5	–1.87768510066488
	*HSP90AB2P*	Putative heat shock protein HSP 90-β 2	0.789186704382044
	*ATP1A1*	Sodium/potassium-transporting ATPase subunit α-1	0.801241448268459
	*CKB*	Creatine kinase B-type	0.719577745146265
	*YWHAE*	14-3-3 Protein ε	–1.27309904712832
Negative regulation of apoptotic signaling pathway			–4.41966
	*CTNNA1*	Catenin α-1	1.22595354036506
	*CTNNB1*	Catenin β-1	0.933497102511857
	*HSPB1*	Heat shock protein β-1	–1.8304341140152
	***MIF***	**Macrophage migration inhibitory factor**	**–0.769452650396329**
	*ACAA2*	3-Ketoacyl-CoA thiolase, mitochondrial	–0.893276481158282
	*BSG*	Basigin	0.809002774939086
	***GRN***	**Granulin**	**–0.928264784720231**
	*USP5*	Ubiquitin carboxyl-terminal hydrolase 5	–0.92420726221496
	*SMARCE1*	SWI/SNF-related matrix-associated actin-dependent regulator of chromatin subfamily E member 1	–0.834904881758245
Regulation of cellular catabolic process			–2.2796
	*HSPB1*	Heat shock protein β-1	–1.8304341140152
	*SEC22B*	Vesicle-trafficking protein SEC22b	–1.08262171411317
	*USP5*	Ubiquitin carboxyl-terminal hydrolase 5	–0.92420726221496
	*FABP1*	Fatty acid–binding protein, liver	–0.806341784614256
Regulation of cellular response to stress			–2.42511
	***GRN***	**Granulin**	**–0.928264784720231**
	*HSPB1*	Heat shock protein β-1	–1.8304341140152
	***MIF***	**Macrophage migration inhibitory factor**	**–0.769452650396329**
	*SMARCE1*	SWI/SNF-related matrix-associated actin-dependent regulator of chromatin subfamily E member 1	–0.834904881758245
Positive regulation of response to external stimulus			–2.09487
	***GRN***	**Granulin**	**–0.928264784720231**
	*HSPB1*	Heat shock protein β-1	–1.8304341140152
	***MIF***	**Macrophage migration inhibitory factor**	**–0.769452650396329**

CXCL8-regulatory proteins are shown in bold. Processes of interests were determined by relevance to stress or immune responses.

KO, knockout; SWI/SNF, SWItch/sucrose non-fermentable; WT, wild type.

**Figure 7:**
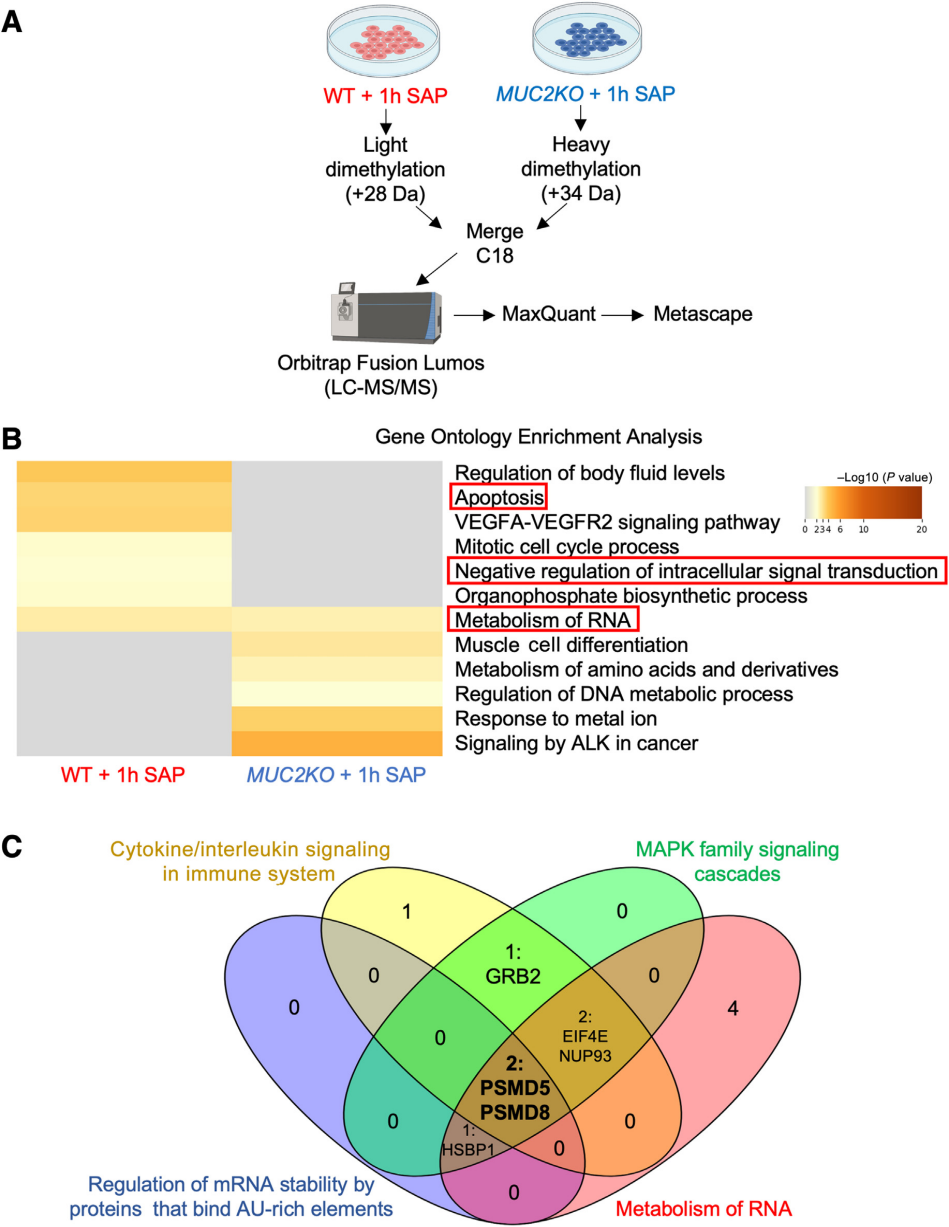
Pathways involved in cytokine signaling and regulation of mRNA are enriched in wild-type (WT) goblet-like cells treated with soluble amebic protein (SAP) compared with *MUC2KO* cells. **A:** Schematic of the sample preparation and workflow for shotgun proteomic analysis. Figure generated with BioRender.com (Toronto, ON, Canada). **B:** Metascape analysis showing Gene Ontology enrichment analysis of WT and *MUC2KO* cells treated with SAP, 100 μg/mL, 1 hour. Pathways of interest are in **red boxed areas**. **C:** Venn diagram highlighting common interesting proteins involved in pathways of interest. Numbers refer to protein hits; proteins in pathways with overlapping sections listed out. Pathways of interests were determined by relevance to stress or immune responses. Venn diagram generated with Venny 2.0 (*https://bioinfogp.cnb.csic.es/tools/venny*). ALK, anaplastic lymphoma kinase; AU, adenylate-uridylate; GRB2, growth factor receptor-bound protein 2; EIF4E, eukaryotic translation initiation factor 4E; HSPB1, heat shock protein beta-1; LC-MS/MS, liquid chromatography–tandem mass spectrometry; MAPK, mitogen-activated protein kinase; NUP93, nucleoporin 93; PSMD, 26S proteasome non-ATPase regulatory subunit; VEGFA, vascular endothelial growth factor-A; VEGFR, vascular endothelial growth factor receptor.

Because shotgun proteomic analysis suggested that WT cells were primed basally to respond to stressors, the pathways that were differentially enriched when stimulated with SAP for 1 hour were determined (Figure 7A and Supplemental Table S2). Gene Ontology enrichment analysis revealed that following SAP treatment, WT cells were enriched in pathways involved in negative regulation of intracellular signal transduction and metabolism of RNA (Figure 7B). WT cells were enriched in pathways involved in cytokine/IL signaling in the immune system, MAPK family signaling cascades, and regulation of mRNA stability by proteins that bind adenylate-uridylate–rich elements (Table 2). This was suggestive of why WT secreted significantly more CXCL8 compared with *MUC2KO* cells when stimulated with *E. histolytica* (Figure 2A). Analysis of the specific protein hits involved in pathways of interest revealed that 26S proteasome non-ATPase regulatory subunit 5 (PSMD5) and PSMD8 were enriched in WT cells (Figure 7C). PSMD5 and PSMD8 are chaperone proteins involved in the assembly of the 26S proteasome, the main proteolytic machine responsible for regulated protein degradation in eukaryotic cells involved in homeostasis, stress responses, and signal transduction.^31^ This suggested that the maintenance of the ubiquitin-proteasome system responsible for regulating cellular processes is disrupted in *MUC2KO* cells that are unable to synthesize mucins. This subsequently interrupts the ability of these cells to secrete other molecules, such as proinflammatory cytokines, to participate in host defense by alternate means.

**Table 2:** Attractive Protein Hits Identified in Pathways of Interest in WT and *MUC2KO* Cells Following 1-Hour Stimulation with SAP

Pathway	Gene symbol	Protein name	Log2 (KO:WT)
Regulation of mRNA stability by proteins that bind AU-rich elements			–8.781658545
	***HSPB1***	**Heat shock protein β-1**	**–1.357566432**
	***PSMD5***	**Proteasome 26S subunit, non-ATPase 5**	**–1.63110875472744**
	***PSMD8***	**Proteasome 26S subunit, non-ATPase 8**	**–1.21752433453817**
Cytokine signaling in immune system			–2.994010194
	***BRWD1***	**Bromodomain andWDrepeat domain-containing 1**	**–2.0835080688084**
	***PSMD5***	**Proteasome 26S subunit, non-ATPase 5**	**–1.63110875472744**
	***EIF4E***	**Eukaryotic translation initiation factor 4E**	**–1.2557355508751**
	***PSMD8***	**Proteasome 26S subunit, non-ATPase 8**	**–1.21752433453817**
	*GRB2*∗	Growth factor receptor-bound protein 2	1.29478246869807
	*NUP93*∗	Nucleoporin 93	2.08031656036671
MAPK family signaling cascades			-2.752425454
	***PSMD5***	**Proteasome 26S subunit, non-ATPase 5**	**–1.63110875472744**
	***HSPB1***	**Heat shock protein β-1**	**–1.357566432**
	***PSMD8***	**Proteasome 26S subunit, non-ATPase 8**	**–1.21752433453817**
	*GRB2*∗	Growth factor receptor-bound protein 2	1.29478246869807
Signaling by ILs			–2.270454087
	***BRWD1***	**Bromodomain and WD repeat domain-containing 1**	**–2.0835080688084**
	***PSMD5***	**Proteasome 26S subunit, non-ATPase 5**	**–1.63110875472744**
	***PSMD8***	**Proteasome 26S subunit, non-ATPase 8**	**–1.21752433453817**
Regulation of translation			–2.18950209
	***EIF3H***	**Eukaryotic translation initiation factor 3 subunit H**	**–1.25953003108888**
	***EIF4E***	**Eukaryotic translation initiation factor 4E**	**–1.2557355508751**
	***HSPB1***	**Heat shock protein β-1**	**–1.357566432**
Metabolism of RNA			–4.802932946
	***PSMD5***	**Proteasome 26S subunit, non-ATPase 5**	**–1.63110875472744**
	***HSPB1***	**Heat shock protein β-1**	**–1.357566432**
	***EIF4E***	**Eukaryotic translation initiation factor 4E**	**–1.2557355508751**
	***PSMD8***	**Proteasome 26S subunit, non-ATPase 8**	**–1.21752433453817**
	*CTNNBL1*∗	β-Catenin–like protein 1	1.1895399443885
	*BUD31*∗	Protein BUD31 homolog	1.24628646399215
	*RRP36*∗	Ribosomal RNA processing protein 36 homolog	1.75232042845967
	*NUP93*∗	Nucleoporin 93	2.08031656036671
	*RPRD1A*∗	Regulation of nuclear pre-mRNA domain-containing protein 1A	2.83157327060919

Proteins enriched in WT cells are shown in bold. Processes of interests were determined by relevance to stress or immune responses.

∗Proteins enriched in *MUC2KO* cells.

AU, adenylate-uridylate; KO, knockout; MAPK, mitogen-activated protein kinase; SAP, soluble amebic protein; WD, beta-transducin; WT, wild type.

**Figure 8:**
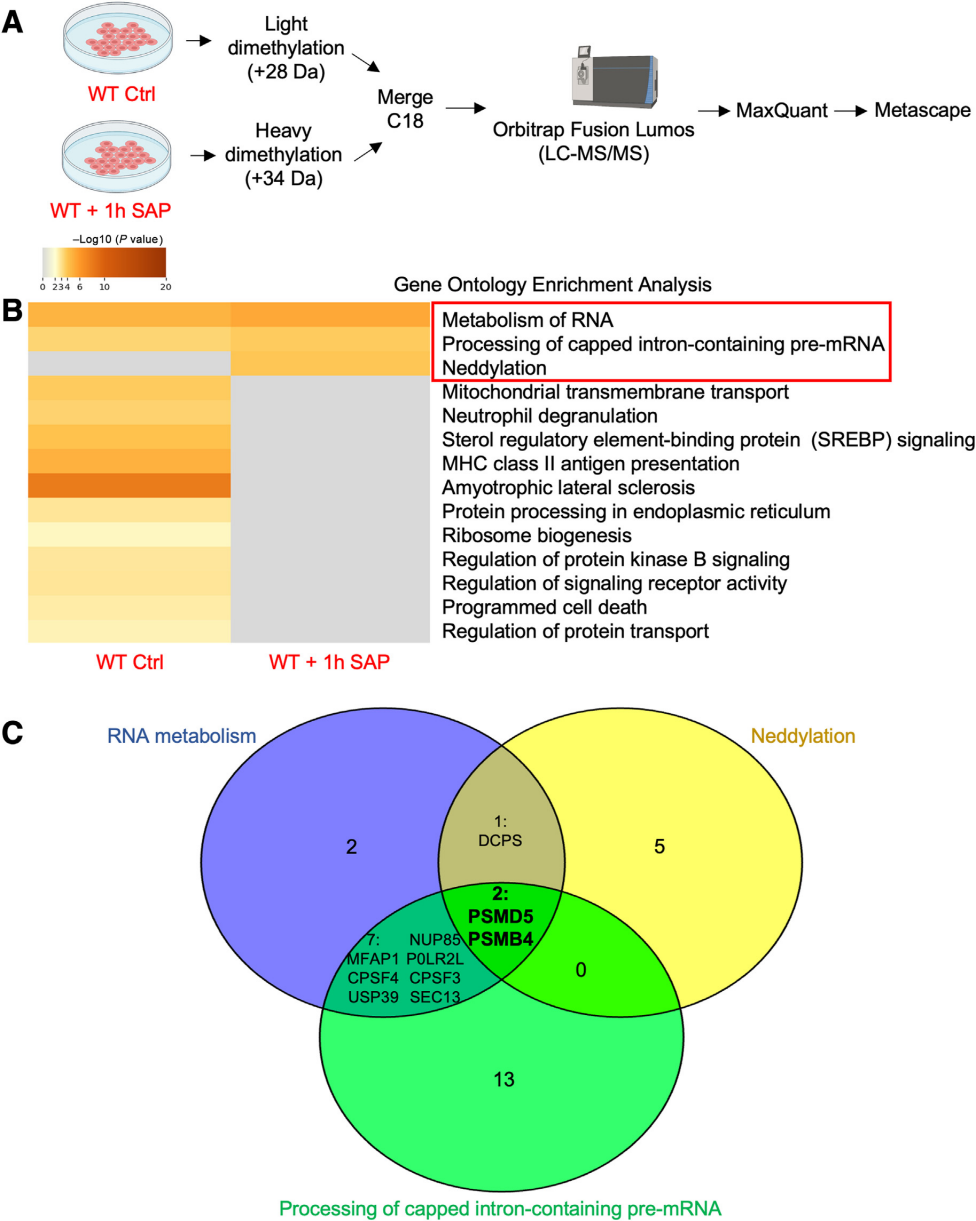
Soluble amebic protein (SAP) treatment enriches pathways involved in RNA metabolism in wild-type (WT) goblet-like cells. **A:** Schematic of the sample preparation and workflow for shotgun proteomic analysis. Figure generated with BioRender.com (Toronto, ON, Canada). **B:** Metascape analysis showing Gene Ontology enrichment analysis of WT goblet cells treated with SAP (100 μg/mL; 1 hour). Pathways of interest are in **red boxed areas**. **C:** Venn diagram highlighting common interesting proteins involved in pathways of interest. Numbers refer to protein hits; proteins in pathways with overlapping sections listed out. Pathways of interests were determined by relevance to stress or immune responses. Venn diagram generated with Venny 2.0 (*https://bioinfogp.cnb.csic.es/tools/venny*). CPSF4, cleavage and polyadenylation specificity factor subunit 3; Ctrl, control; DCPS, decapping scavenger enzyme; LC-MS/MS, liquid chromatography–tandem mass spectrometry; MFAP1, microfibrillar-associated protein 1; MHC, major histocompatibility complex; NUP85, nucleoporin 85; POLR2L, DNA-directed RNA polymerases I, II, and III subunit RPABC5; PSMD, 26S proteasome non-ATPase regulatory subunit; SEC13, SEC13 homolog, nuclear pore and COPII coat complex component; USP39, ubiquitin carboxyl-terminal hydrolase 39.

**Figure 9:**
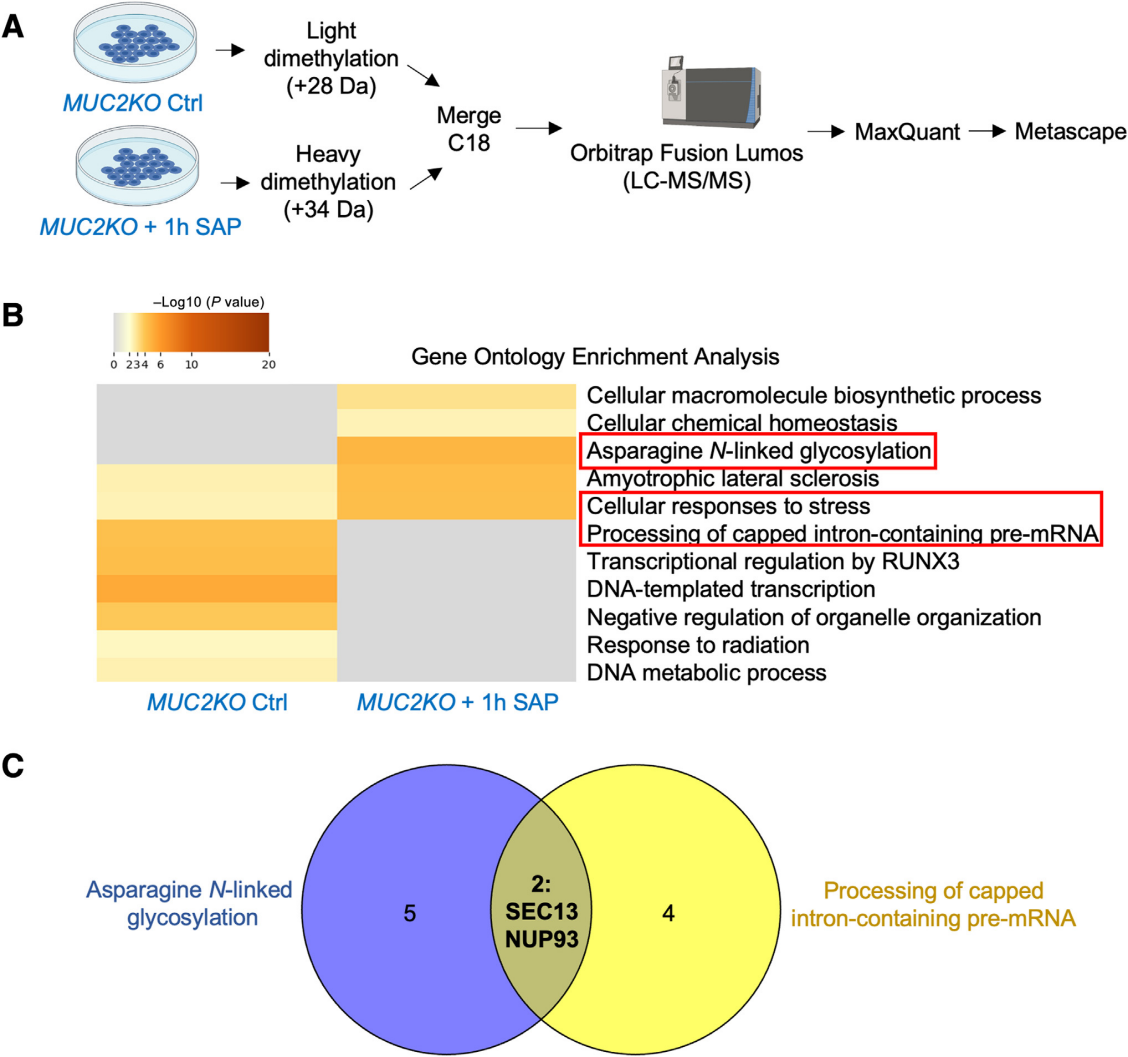
Soluble amebic protein (SAP) treatment enriches pathways involved in glycosylation and response to stress pathways in *MUC2KO* goblet-like cells. **A:** Schematic of the sample preparation and workflow for shotgun proteomic analysis. Figure generated with BioRender.com (Toronto, ON, Canada). **B:** Metascape analysis showing Gene Ontology enrichment analysis of *MUC2KO* cells treated with SAP (100 μg/mL; 1 hour). Pathways of interest are in **red boxed areas**. **C:** Venn diagram highlighting common interesting proteins involved in pathways of interest. Numbers refer to protein hits; proteins in pathways with overlapping sections listed out. Pathways of interests were determined by relevance to stress or immune responses. Venn diagram generated with Venny 2.0 (*https://bioinfogp.cnb.csic.es/tools/venny*). Ctrl, control; LC-MS/MS, liquid chromatography–tandem mass spectrometry; NUP93, nucleoporin 93; RUNX3, runt-related transcription factor 3; SEC13, SEC13 homolog, nuclear pore and COPII coat complex component.

To determine what pathways of interest were up-regulated in response to *E. histolytica*, WT cells basally, and with 1 hour of SAP treatment were compared, as were *MUC2KO* cells basally, and with 1 hour of SAP treatment by shotgun proteomics (Figures 8A and 9A and Supplemental Tables S3 and S4). In response to SAP, WT cells were enriched in pathways involved in metabolism of RNA, processing of capped intron-containing pre-mRNA, and neddylation (Figure 8B). PSMD5 and PSMB4 proteins were common in these pathways (Figure 8C and Table 3). This was intriguing as the previous analysis comparing WT and *MUC2KO* cells treated with SAP found that PSMD5, a chaperone protein involved in the assembly of the 26S proteasome, was enriched in WT cells (Figure 7C). This indicated that *E. histolytica* induced the expression of this protein in WT cells from basal states, as well as in the absence of mucin production. The enrichment of neddylation in WT cells in response to SAP treatment was also interesting, as recent studies have shown that this process regulates NF-κβ–dependent proinflammatory cytokine release from macrophages in response to *E. histolytica* by silencing the proteins cullin-1/5 to inhibit NF-κβ signaling.^32^ Neddylation is a post-translational modification that adds the ubiquitin-like protein neural precursor cell expressed developmentally downregulated protein 8 (NEDD8) to lysine residues of substrate proteins to modulate various biological processes by affecting the protein's stability, subcellular localization, conformation, or function.^33^ In contrast, in *MUC2KO* cells, asparagine N-linked glycosylation was enriched, whereas processing of capped intron-containing pre-mRNA was down-regulated in response to SAP (Figure 9B and Table 4). Comparison of common proteins in these pathways identified SEC13 homolog, nuclear pore and COPII coat complex component (SEC13), which was down-regulated in response to SAP, and nucleoporin 93 (NUP93), which was up-regulated in response to SAP (Figure 9C). Asparagine *N*-linked glycosylation is another post-translational protein modification that is involved in molecular recognition, protein folding, sorting in the ER, cell-to-cell communication, and protein stability.^34^ *N*-glycosylation can also serve to flag misfolded proteins for proteasomal degradation.^34^ As SAP could induce ROS production in *MUC2KO* cells (Figure 1D), enrichment of this pathway in response to *E. histolytica* in *MUC2KO* cells was not surprising, as this is a way by which these cells respond to increased demand on the ER.

**Table 3:** Attractive Protein Hits Identified in Pathways of Interest in LS174T WT Cells Following 1-Hour SAP Stimulation

Pathway	Gene symbol	Protein name	Log2 (WT:SAP)
Metabolism of RNA			
	*MFAP1*	Microfibril-associated protein 1	2.539928043
	*CPSF4*	Cleavage and polyadenylation–specific factor 4	1.45016869730833
	*PSMD5*	Proteasome 26S subunit, non-ATPase 5	1.21368944375248
	*USP39*	Ubiquitin-specific peptidase 39	1.13940400580658
	*DCPS*	Decapping enzyme, scavenger	1.119953718
	*NUP85*	Nucleoporin 85	–1.51259597511386
	*PSMB4*	Proteasome 20S subunit β 4	–1.54654036679693
	*POLR2L*	RNA polymerase II, I, and III subunit L	–1.58078473993205
	*HEATR1*	HEAT repeat-containing 1	–1.813753014
	*CPSF3*	Cleavage and polyadenylation–specific factor 3	–1.9403678401179
	*NOC4L*	Nucleolar complex–associated 4 homologs	–2.08914456076491
	*SEC13*	SEC13 homolog, nuclear pore and COPII coat complex component	–2.67291052975576
Neddylation			
	*LGMN*	Legumain	3.04472633814415
	*UCHL3*	Ubiquitin carboxyl-terminal hydrolase; ubiquitin carboxyl-terminal hydrolase isozyme L3	1.79630702627434
	*CUL4A*	Cullin-4A	1.339023333
	*PSMD5*	Proteasome 26S subunit, non-ATPase 5	1.21368944375248
	*DCPS*	Decapping enzyme, scavenger	1.119953718
	*PSMB4*	Proteasome 20S subunit β 4	–1.54654036679693
	*UBE2L3*	Ubiquitin-conjugating enzyme E2 L3	–2.23317589087678
	*DNAJC10*	DnaJ homolog subfamily C member 10	–2.02691042973606
Processing of capped intron-containing pre-mRNA			
	*MFAP1*	Microfibril-associated protein 1	2.539928043
	*CPSF4*	Cleavage and polyadenylation specificity factor subunit 4	1.45016869730833
	*PSMD5*	Proteasome 26S subunit, non-ATPase 5	1.21368944375248
	*USP39*	U4/U6.U5 tri-snRNP–associated protein 2	1.13940400580658
	*NUP85*	Nucleoporin 85	–1.51259597511386
	*PSMB4*	Proteasome 20S subunit β 4	–1.54654036679693
	*PRELP*	Prolargin	–1.56149422021725
	*POLR2L*	RNA polymerase II, I, and III subunit L	–1.58078473993205
	*TOMM40*	Mitochondrial import receptor subunit TOM40 homolog	–1.63823164058085
	*AP1G1*	AP-1 complex subunit γ-1	–1.65185824786992
	*ARPC5*	Actin-related protein 2/3 complex subunit 5	–1.7345631039509
	*LRRC1*	Leucine-rich repeat-containing protein 1	–1.74168611235078
	*DDOST*	Dolichyl-diphosphooligosaccharide–protein glycosyltransferase 48-kDa subunit	–1.74482560965285
	*RRM2*	Ribonucleoside-diphosphate reductase subunit M2	–1.78483086251976
	*DNAJC2*	DnaJ homolog subfamily C member 2; DnaJ homolog subfamily C member 2, N-terminally processed	–1.80455160554549
	*CPSF3*	Cleavage and polyadenylation specificity factor subunit 3	–1.9403678401179
	*MYDGF*	Myeloid-derived growth factor	–1.96370064487866
	*PPP2R5B*	Serine/threonine-protein phosphatase 2A 56-kDa regulatory subunit β isoform	–1.97749724580795
	*C1QBP*	Complement component 1 Q subcomponent-binding protein, mitochondrial	–2.34945599711064
	*TUBB6*	Tubulin β-6 chain	–2.51276063813303
	*SEC13*	SEC13 homolog, nuclear pore and COPII coat complex component	–2.67291052975576

Proteins enriched in control WT cells are negative, and proteins enriched in SAP-treated cells are positive. Processes of interests were determined by relevance to stress or immune responses.

AP-1, activating protein-1; HEAT, Huntington, elongation factor 3, PR65/A, TOR; SAP, soluble amebic protein; snRNP, small nuclear ribonucleoprotein; TOM40, translocase of outer mitochondrial membrane 40; WT, wild type.

**Table 4:** Attractive Protein Hits Identified in Pathways of Interest in *MUC2KO* Cells Following 1-Hour SAP Stimulation

Pathway	Gene symbol	Protein name	Log2 (*MUC2KO*:SAP)
Asparagine N-linked glycosylation			
	*SEC31A*	Protein transport protein Sec31A	2.93479954021123
	*ARF1*	ADP-ribosylation factor 1; ADP-ribosylation factor 3	1.65622176328646
	*UGGT1*	UDP-glucose:glycoprotein glucosyltransferase 1	1.65539762772816
	*SEC13*	Protein SEC13 homolog	1.40621010853157
	*SPTA1*	Spectrin α chain, erythrocytic 1	–1.29275794495606
	*TPD52*	Tumor protein D52	–1.34303691701789
	*NUP93*	Nuclear pore complex protein Nup93	–2.42499754313449
Processing of capped intron-containing pre-mRNA			
	*SEC13*	Protein SEC13 homolog	1.40621010853157
	*SRSF5*	Serine/arginine-rich splicing factor 5	–1.1624603656206
	*PRPF4*	U4/U6 small nuclear ribonucleoprotein Prp4	–1.1462118923486
	*PSMD9*	26S proteasome non-ATPase regulatory subunit 9	–1.27100853785212
	*PHF5A*	PHD finger-like domain-containing protein 5A	–1.493865255092
	*NUP93*	Nuclear pore complex protein Nup93	–2.42499754313449

Proteins enriched in control *MUC2KO* cells are negative, and proteins enriched in SAP-treated cells are positive. Processes of interests were determined by relevance to stress or immune responses.

PHD, plant homeodomain; SAP, soluble amebic protein.

### ER Stress Drives the Stability of CXCL8 Transcripts Produced in Response to *E. histolytica*

**Figure 10:**
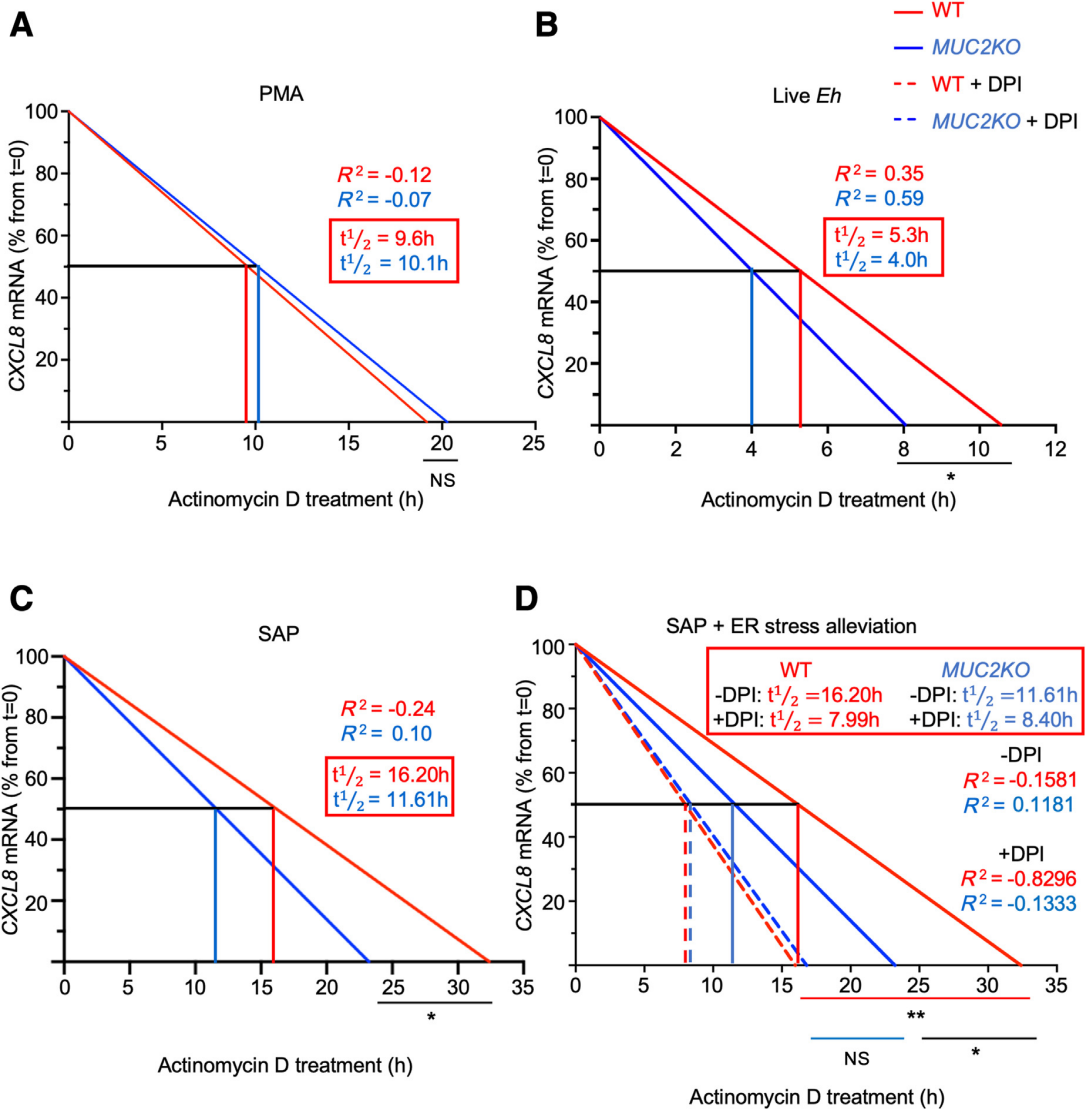
The stability of CXCL8 transcripts produced in response to *Entamoeba histolytica* by wild-type (WT) goblet-like cells is dependent on endoplasmic reticulum (ER) stress. **A**–**C:** Half-lives of CXCL8 transcripts as determined by linear regression analysis with actinomycin D treatment (10 μg/mL) following phorbol 12-myristate 13-acetate (PMA) stimulation, 1 μmol/L, 2 hours, live *E. histolytica* stimulation, 40,000 trophozoites per well, 1 hour, or soluble amebic protein (SAP) stimulation, 100 μg/mL, 1 hour. **D:** Half-lives of CXCL8 transcripts as determined by linear regression analysis with actinomycin D treatment (10 μg/mL) following SAP stimulation, 100 μg/mL, 1 hour, with or without diphenyleneiodonium chloride (DPI) pretreatment, 10 μmol/L, 30 minutes. Half-lives of transcripts are in **red boxed areas**. mRNA was taken at 0, 0.5, 1, 2, 4, 6, 8, 12, and 24 hours after actinomycin D treatment. mRNA was quantified through quantitative RT-PCR, and linear regression analysis was used to calculate mRNA half-lives. Slopes were compared by analysis of covariance. **A**–**D:** Data are from three independent experiments with three to four individual samples per time point. *n* = 9 to 12. ∗*P* < 0.05, ∗∗*P* < 0.01. NS, not significant.

**Figure 11:**
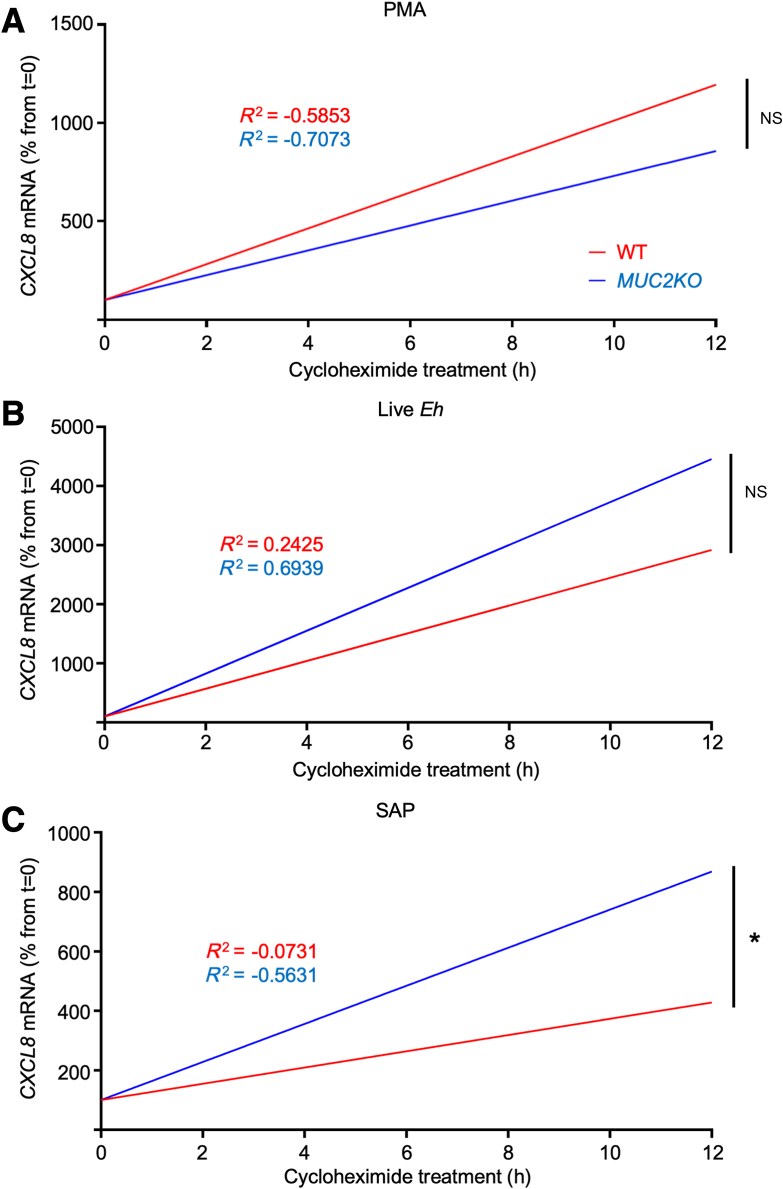
CXCL8 transcript degradation is dependent on newly synthesized proteins. **A**–**C:** CXCL8 transcripts produced as determined by linear regression analysis with cycloheximide treatment (10 μg/mL) following phorbol 12-myristate 13-acetate (PMA) stimulation, 1 μmol/L, 2 hours, live *Entamoeba histolytica* stimulation, 40,000 trophozoites per well, 1 hour, or soluble amebic protein (SAP) stimulation, 100 μg/mL, 1 hour. mRNA was taken at 0, 0.5, 1, 2, 4, 6, 8, 12, and 24 hours after cycloheximide treatment. mRNA was quantified through quantitative RT-PCR, and linear regression analysis was used to determine degradation of transcripts. Slopes were compared by analysis of covariance. **A**–**C:** Data are from three independent experiments with three to four individual samples per time-point. *n* = 9 to 12. ∗*P* < 0.05. NS, not significant; WT, wild type.

**Supplemental Figure S1:**
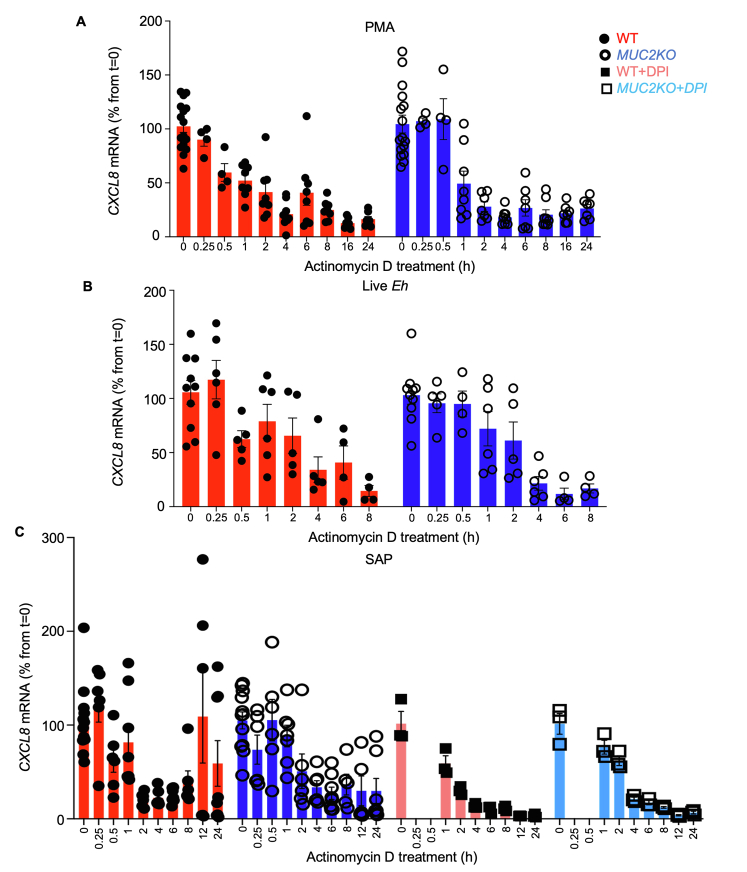
Real-time quantitative PCR (qPCR) data demonstrating the stability of *CXCL8* transcripts produced in response to *Entamoeba histolytica* by wild-type (WT) goblet-like cells is dependent on endoplasmic reticulum stress. **A** and **B:** qPCR data of *CXCL8* of WT and *MUC2KO* cells after actinomycin D treatment (10 μg/mL) following phorbol 12-myristate 13-acetate (PMA) stimulation, 1 μmol/L, 2 hours, or live *E. histolytica* stimulation, 40,000 trophozoites per well, 1 hour mRNA was taken at 0, 0.5, 1, 2, 4, 6, 8, 12, and 24 hours after actinomycin D treatment. **C:** qPCR data of *CXCL8* of WT and *MUC2KO* cells after actinomycin D treatment (10 μg/mL) following soluble amebic protein (SAP) stimulation, 100 μg/mL, 1 hour, with or without diphenyleneiodonium chloride (DPI) pretreatment, 10 μmol/L, 30 minutes. mRNA was taken at 0, 0.5, 1, 2, 4, 6, 8, 12, and 24 hours after actinomycin D treatment. **A**–**C:** Data are from three independent experiments with three to four individual samples per time point. *n* = 9 to 12.

**Supplemental Figure S2:**
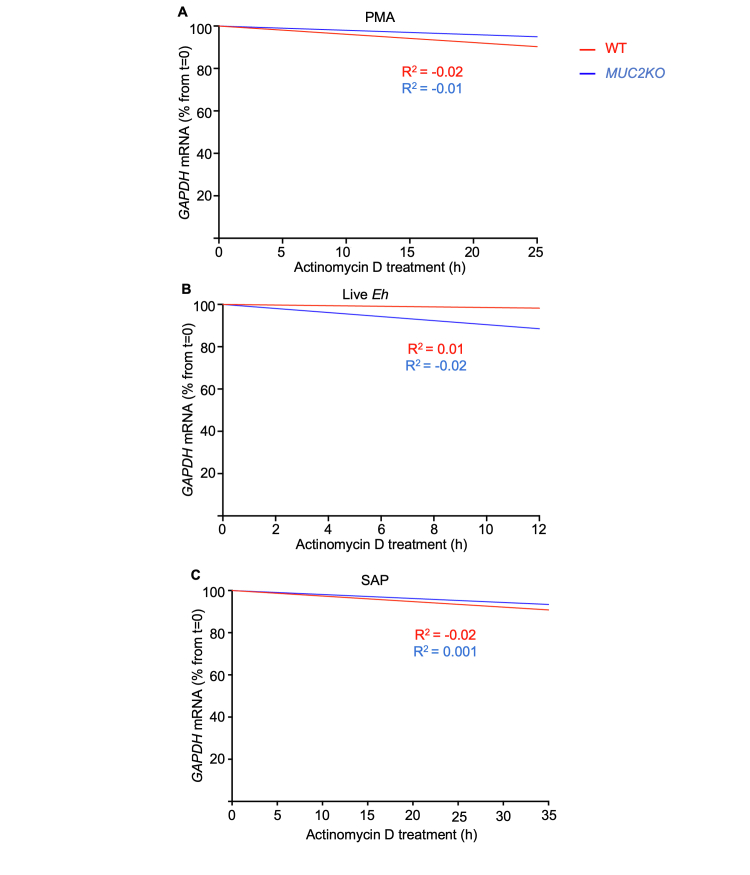
The stability of *GAPDH* transcripts produced in response to *Entamoeba histolytica* (*Eh*) by wild-type (WT) goblet cells is independent of endoplasmic reticulum stress. **A**–**C:** Half-lives of *GAPDH* transcripts as determined by linear regression analysis with actinomycin D treatment (10 μg/mL) following phorbol 12-myristate 13-acetate (PMA) stimulation, 1 μmol/L, 2 hours, live *E. histolytica* stimulation, 40,000 trophozoites per well, 1 hour, or soluble amebic protein (SAP) stimulation, 100 μg/mL, 1 hour.

Proteomic analysis suggested that pathways involved in mRNA stability were enriched in WT compared with those in *MUC2KO* cells following amebic stimulation. Therefore, the differences in the stabilization of CXCL8 transcripts produced by the two cell lines in response to *E. histolytica* were explored. To do so, WT and *MUC2KO* cells were stimulated with PMA, live *E. histolytica*, or SAP to induce maximal CXCL8 transcription, before actinomycin D was added to inhibit transcription. The fates of the transcripts were followed over time by real-time quantitative PCR, with maximal transcription representing 100% mRNA at 0 hours of actinomycin D treatment. Linear regression analysis was used to calculate the half-lives of transcripts produced (Figure 10, A–C, and Supplemental Figure S1). Following stimulation with PMA, no significant differences in the half-lives of CXCL8 transcripts was observed between the cells (Figure 10A). In marked contrast, when WT and *MUC2KO* cells were treated with either live *E. histolytica* or SAP, CXCL8 transcripts from WT cells had significantly longer half-lives compared with those from *MUC2KO* cells, indicating these transcripts were more stable (Figure 10, B and C). In particular, the half-life of CXCL8 transcripts treated with SAP in WT was 16.20 hours compared with 11.61 hours in *MUC2KO* cells. To determine whether ROS production during MUC2 mucin biosynthesis was driving the stability of CXCL8 transcripts, cells were pretreated with DPI to alleviate ER stress. Remarkably, in the presence of DPI, the half-lives of CXCL8 transcripts were reduced from both cell lines but only significantly so in WT cells [half-life (t½) 16.20 versus 7.99 hours in the presence of DPI], revealing that ER stress affected the stability of CXCL8 transcripts produced in response to SAP (Figure 10D). To confirm that the reduction of half-lives was specific to CXCL8 transcription, the control gene, GAPDH, was analyzed and no degradation was observed, as expected (Supplemental Figure S2). To determine whether endogenous proteins were differentially degrading CXCL8 transcripts between the cell lines, cells were stimulated with PMA, live *E. histolytica*, or SAP, and cycloheximide was added to inhibit translation of new proteins. Regardless of the stimulus, there was an accumulation of CXCL8 transcripts in the cells, revealing that the degradation of the transcripts was dependent on newly synthesized proteins (Figure 11). Based on the results of this study, a model by which mucin-producing goblet-like cells participate in innate host defense against *E. histolytica* by secreting the chemokine, CXCL8, was proposed. These data indicated that maximal proinflammatory responses in goblet-like cells are dependent on ER stress driven by MUC2 mucin biosynthesis and ROS production that activates multiple proinflammatory signaling pathways.

## Discussion

Although the role of colonic goblet cells in innate host defense is often viewed as only a mucus producer, recent studies have revealed that goblet cells produce a variety of other proteins that are involved in immunity, suggesting that goblet cells have pleotropic innate immune functions.^35^^,^^36^ A major deficiency in this field is whether proinflammatory cytokines/chemokines are also produced by colonic goblet cells, and its significance in parasitic infections. As mucin hypersecretion and robust proinflammatory responses are the first indicators of symptomatic *E. histolytica* infections, it stands to reason that goblet cells are central mediators of these responses. The focus of this study was to elucidate the mechanisms by which proinflammatory cytokine responses are regulated in colonic goblet-like cells in response to *E. histolytica.* The major findings of this study revealed that in response to *E. histolytica* and SAP, metabolically stressful MUC2 biosynthesis and production augmented CXCL8 expression and secretion from colonic goblet-like cells via multiple proinflammatory signaling pathways, and this process was disrupted in goblet-like cells unable to produce MUC2.

In this study, LS174T, an *in vitro* model of goblet-like cells, and CRISPR-Cas9 *MUC2KO* cells were used to investigate the impact of mucin biosynthesis and production on proinflammatory cytokine release. At present, primary or immortalized human goblet cells do not exist. The LS174T cell line was obtained from cells originally isolated from the colon of a female adenocarcinoma patient with colorectal cancer. It has long been used as a model for colonic goblet cells because of its epithelial morphology, ability to synthesize and secrete large amounts of mature MUC2, and the presence of mucin granules in its thecae.^37^^,^^38^ The *MUC2KO* cell line was previously generated by transfecting LS174T cells with a plasmid containing MUC2-specific guided-RNA sequences and selectively cloned. The clones were then screened by PCR to confirm the successful deletion of the MUC2 gene.^12^ However, it is important to consider that the CRISPR-Cas9 systems have some limitations, such as the potential of off-target effects that may result in the selection process.^39^ To control for potential off-target effects, all statistical analyses were conducted comparing each cell line with its own unstimulated controls when treated with *E. histolytica*.

Goblet cells hypersecrete mucus as a first line of innate host defense against *E. histolytica*. *E*. *histolytica* binds to galactose and *N*-acetyl D-galactosamine residues on MUC2 mucins via their specific galactose and *N*-acetyl D-galactosamine lectins, and this prevents direct amebic attachment to underlying epithelial cells.^40^ To disrupt the mucus layer, *E. histolytica* uses its cysteine protease, *E. histolytica* CP-A5, to cleave MUC2 at the C-terminus, which also induces mucus hypersecretion from goblet cells.^41^^,^^42^ Mucin hypersecretion can also be driven by *E. histolytica*–derived prostaglandin E2 via activation of EP4 receptors on goblet cells.^43^ As mucin hypersecretion leads to depletion of mucin stores, and subsequent disruption of the mucus barrier, this is a mechanism by which *E. histolytica* disrupts host defenses to drive disease pathogenesis. Previous studies have shown that colonic columnar epithelial cells produce the proinflammatory chemokine, CXCL8, in response to *E. histolytica*, independent of direct cell-to-cell contact, regulated in part by amebic prostaglandin E_2_. However, whether this applies to goblet cells is not known.^8^^,^^9^ Here, the secretion of CXCL8 was an early response by goblet-like cells to recruit innate immune cells to fight against *E. histolytica* infection, which is not dependent on direct parasite-to-epithelial cell contact.

Deficiencies in MUC2 biosynthesis reduce ER stress and cellular autophagy at basal states.^12^ This was driven by higher ROS production basally in WT goblet cells, which was increased following *E. histolytica* stimulation. Investigation of the proinflammatory cytokines revealed that CXCL8 was the dominant proinflammatory mediator secreted by WT goblet cells compared with *MUC2KO* in response to *E. histolytica*. Shotgun proteomic analysis of WT and *MUC2KO* cells basally, as well as in response to SAP, revealed that the differences in proinflammatory phenotype could be explained by priming of basal WT goblet cells by CXCL8-regulatory proteins, such as GRN and MIF, and activation of multiple cytokine and proinflammatory signaling pathways in WT cells. Moreover, analysis of specific signaling pathways confirmed that the MAPK/ERK, MAPK/p38, PI3K/Akt, MAPK/p38, and NF-κβ/IκBα pathways were involved in CXCL8 regulation in WT cells. On the other hand, only the MAPK/ERK and NF-κβ/IκBα pathways regulated CXCL8 in *MUC2KO* goblet cells in response to *E. histolytica*. Activation of multiple signaling pathways resulted in stabilization of CXCL8 transcripts produced in WT cells, which was disrupted with ER stress alleviation. These results are intriguing as a similar phenotype is observed in acute *in vivo* models of amebiasis. Although mice are unable to produce CXCL8, they do produce the murine homologue, keratinocytes-derived chemokine (KC). *Muc2*^*−/−*^ mice endogenously produced higher levels of KC so no significant differences in KC production were observed between control and *E. histolytica* inoculated in colonic loops. *Muc2*^*+/+*^ littermates produced significantly higher amounts of KC in response to *E. histolytica*–inoculated colonic loops.^44^ These results reveal the importance of MUC2 biosynthesis in driving proinflammatory chemokine responses from goblet cells against *E. histolytica* infection.

ER stress induces ROS production from cells during the unfolded protein response. Proteins such as MUC2, that have more disulfide bonds, lead to greater formation of superoxide anion radicals generated during the formation of these bonds.^45^ ATF4, a marker of the unfolded protein response, is activated by various cell stressors, including ER stress driven by an accumulation of misfolded proteins, hypoxia, and oxidative stress.^46^ ATF4 also functions to induce transcription of downstream proteins involved in the unfolded protein response to either drive resistance to stress by degrading unfolded or misfolded proteins or induce apoptosis of the cell.^47^ ROS induces ATF4 that feeds back into the unfolded protein response signaling pathway to either shift the cell to survival states or toward apoptosis to handle these additional stressors.^48^ Importantly, immune cells produce ROS during *E. histolytica* infections to kill trophozoites. Macrophage activation for cytotoxicity against *E. histolytica* is mediated by nitric oxide, and hydrogen peroxide is important for respiratory bursts from macrophages for *E. histolytica* trophozoite killing.^49^^,^^50^ ROS is required for activation of the nucleotide-binding domain, leucine-rich-containing family, pyrin domain-containing-3 (NLRP3) inflammasome in alveolar macrophages and subsequent secretion of proinflammatory cytokines, IL-1β and IL-18.^51^ These cytokines are also produced in response to *E. histolytica* infections from hyperactivated macrophages.^52^ Neutrophils are also potent ROS generators, which is vital for the clearance of invading microorganisms, and subsequent activation of granular proteases, formation of neutrophil extracellular traps, and stimulation of proinflammatory cytokines, such as tumor necrosis factor-α and macrophage inflammatory protein 2 (MIP-2).^53^ Intriguingly, although neutrophil extracellular trap formation is induced by *E. histolytica* contact, this was through a non-classic mechanism independent of NADPH oxidase 2 (NOX2)–derived ROS and peptidyl arginine deiminase (PAD4) activation.^54^ It is possible that the production of ROS from colonic goblet cells may also serve to activate these innate immune cells in addition to proinflammatory cytokine production.

The results of this study suggest a novel role for colonic goblet-like cells in shaping the proinflammatory landscape in response to *E. histolytica*. However, the true impact of this in an *in vivo* setting with other cell types contributing to inflammation must be investigated further. Intestinal epithelial cells and innate immune cells such as macrophages release robust levels of proinflammatory cytokines during symptomatic *E. histolytica* infection, which results in subsequent tissue damage and parasitic dissemination. *E*. *histolytica* may induce mucus hypersecretion to target goblet cells by diminishing immune cell infiltration to drive disease pathogenesis. It is curious that CXCL8 appeared to be the dominant cytokine released by goblet cells even though symptomatic amebic infections include elevated levels of other cytokines, such as IL-1β, IL-6, IL-12, interferon--γ, and tumor necrosis factor-α.^16^ Why this proinflammatory phenotype is observed in goblet cells compared with other intestinal epithelial cells warrants further investigation. Overall, the findings of this study uncovered a potential novel role that mucin-producing colonic goblet-like cells play during innate host defense by secreting proinflammatory chemokines to recruit innate immune cells against *E. histolytica* infection.

## Disclosure Statement

None declared.
